# Functional annotation of Alzheimer's disease associated loci revealed by GWASs

**DOI:** 10.1371/journal.pone.0179677

**Published:** 2017-06-26

**Authors:** Zengpeng Han, Han Huang, Yue Gao, Qingyang Huang

**Affiliations:** School of Life Sciences, Central China Normal University, Wuhan, China; Nathan S Kline Institute, UNITED STATES

## Abstract

Genome-wide association studies (GWASs) discovered a number of SNPs and genes associated with Alzheimer's disease (AD). However, how these SNPs and genes influence the liability to AD is not fully understood. We deployed computational approaches to explore the function and action mechanisms of AD -related SNPs and genes identified by GWASs, including the effects of 195 GWAS lead SNPs and 338 proxy SNPs on miRNAs binding and protein phosphorylation, their RegulomeDB and 3DSNP scores, and gene ontology, pathway enrichment and protein-protein interaction network of 126 AD-associated genes. Our computational analysis identified 6 lead SNPs (rs10119, rs1048699, rs148763909, rs610932, rs6857 and rs714948) and 2 proxy SNPs (rs12539172 and rs2847655) that potentially impacted the miRNA binding. Lead SNP rs2296160 and proxy SNPs rs679620 and rs2228145 were identified as PhosSNPs potentially influencing protein phosphorylation. AD-associated genes showed enrichment of “regulation of beta-amyloid formation”, “regulation of neurofibrillary tangle assembly”, “leukocyte mediated immunity” and “protein-lipid complex assembly” signaling pathway. Protein-protein interaction network and functional module analyses identified highly-interconnected “hub” genes (*APOE*, *PICALM*, *BIN1*, *ABCA7*, *CD2AP*, *CLU*, *CR1*, *MS4A4E* and *MS4A6A*) and bottleneck genes (*APOE*, *TOMM40*, *NME8*, *PICALM*, *CD2AP*, *ZCWPW1*, *FAM180B*, *GAB2* and *PTK2B*) that created three tight subnetworks. Our results provided the targets for further experimental assessment and further insight on AD pathophysiology.

## Introduction

Alzheimer disease (AD), the most common neurodegenerative disease and the leading cause of dementia, is characterized by progressive loss of memory and deficits in thinking, problem solving, and communicating. Due to recent improvements in life expectancy, the prevalence of AD has rapidly grown worldwide and is predicted to affect 1 in 85 people globally by 2050 [1]. No preventative or curative treatments are currently available; therefore, AD has become a major public health burden [2]. The etiology of AD is poorly understood; however, genetic factors have shown to play a pivotal role [3]. Since 2005, genome-wide association studies (GWASs) have successfully identified genetic loci that contribute to susceptibility to many complex diseases [4, 5]. At the end of 2015, 126 genes and 195 single nucleotide polymorphisms (lead SNPs) have been associated with AD at the threshold for genome-wide significance (*P* < 5 × 10^−8^) in 38 GWASs and three meta-analyses (http://www.ebi.ac.uk/gwas/home). Elucidating how these SNPs and genes affect AD liability is the next major challenge [4]. Computational approaches are useful for post-GWAS studies aimed at prioritizing potential causal SNPs for experimental investigation. Various computational approaches and tools have been developed for this purpose [6] and experimentally validated by identifying functional genetic variants and previously unknown molecular mechanisms for various complex disorders, including type 2 diabetes [7], obesity [8], and AD [9].

**Fig 1:**
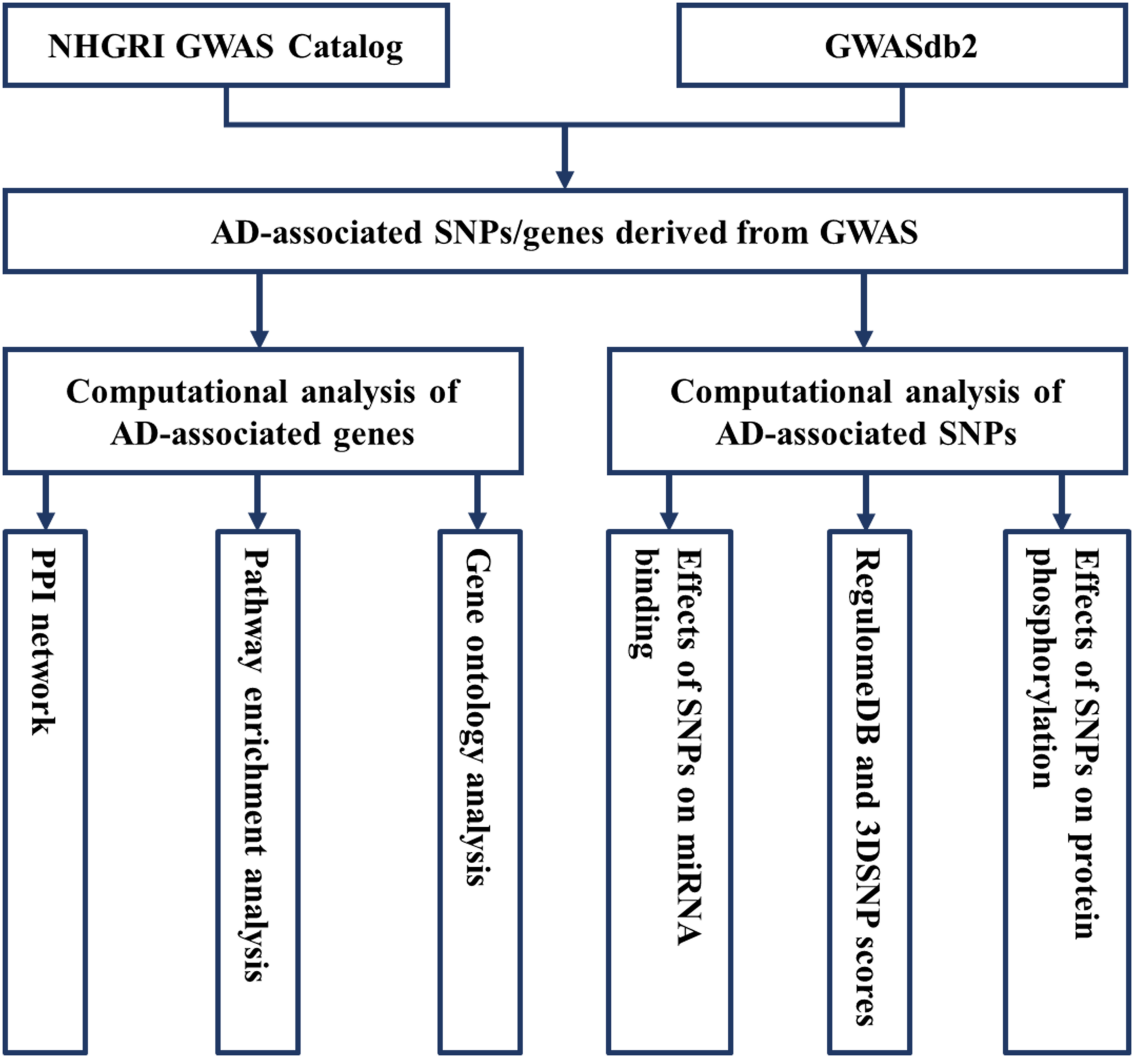
Workflow for Alzheimer’s disease -associated loci derived from genome-wide association studies.

As a starting point of guiding functional study design [6], we recently annotated the function of SNPs and genes for osteoporosis [10] and type 2 diabetes [11] using bioinformatics methods. Rosenthal et al. [12] previously investigated potential regulatory functions of lead SNPs and their proxy SNPs identified in five GWASs of late-onset AD using RegulomeDB. In this study, we predicted the effects of AD GWAS lead SNPs and their proxy SNPs on miRNA binding and protein phosphorylation, evaluated the functionality of SNPs using RegulomeDB and 3DSNP scores, and carried out gene ontology, pathway enrichment, and protein–protein interaction (PPI) network analysis for AD-associated genes (Fig 1). The aims of our study are to identify potential SNPs for follow-up functional analyses and to offer guidance for future research with respect to the etiology and pathogenesis of AD.

## Methods

### Data collection

The National Human Genome Research Institute (NHGRI) catalog of published GWAS provides a publicly available, manually curated collection of published GWAS assaying at least 100000 SNPs and all SNP-trait associations with *P* < 1 × 10^−5^ [5]. The GWASdb provides information of genetic variants except for that annotated in the NHGRI GWAS Catalog and manually curated the SNPs that are marginally significant (*P* < 1.0 × 10^−3^), collected from supplementary materials of each original publication. Using *P* < 5 × 10^−8^ as a significant threshold, we interrogated and downloaded the GWAS association results for AD from the NHGRI GWAS Catalog (http://www.ebi.ac.uk/gwas/home) and GWASdb (v2.0) (http://jjwanglab.org/gwasdb) (updated to the end, 2015). We searched the proxy SNPs, which were in strong linkage disequilibrium (LD) (r^2^ ≥ 0.8) with AD lead SNPs via SNP Annotation and Proxy Search (http://www.broadinstitute.org/mpg/snap/ldsearch.php) [13], based on genotype data from the 1000 Genomes Pilot 1 Project [14] and the International HapMap Project (v3) [15] with the CEU population panel. The analyses were carried out manually, SNP by SNP, with the limits of r^2^ ≥ 0.80 and 500 kb from the query lead SNPs.

### Functional annotation of AD-associated SNPs using RegulomeDB and 3DSNP

RegulomeDB (http://regulomedb.org) and 3DSNP (http://biotech.bmi.ac.cn/3dsnp) are two valuable resources for the annotation of human SNPs. RegulomeBD classifies SNPs into classes based on the combinatorial presence/absence status of functional categories, including transcription factors binding sites, DNAase hypersensitivity regions, and promoter regions [16, Table 1]. Each SNP has been assigned a RegulomeDB score (range: 1–6) to indicate the potential function. In 3DSNP, each SNP is scored based on six functional categories: 3D interacting genes, enhancer state, promoter state, transcription factor binding sites, altered sequence motifs, and conservation score [17]. 3DSNP adopts a quantitative scoring system to measure the functionality of a SNP. All AD-associated lead SNPs and their proxy SNPs (r^2^ ≥ 0.80) have been scored to evaluate the functional significance of SNPs using RegulomeDB and 3DSNP, one by one.

**Table 1:** RegulomeDB score and related functional annotation.

Score	Description
1a	eQTL + TF binding + matched TF motif + matched DNase Footprint + DNase peak
1b	eQTL + TF binding + any motif + DNase Footprint + DNase peak
1c	eQTL + TF binding + matched TF motif + DNase peak
1d	eQTL + TF binding + any motif + DNase peak
1e	eQTL + TF binding + matched TF motif
1f	eQTL + TF binding/DNase peak
2a	TF binding + matched TF motif + matched DNase Footprint + DNase peak
2b	TF binding + any motif + DNase Footprint + DNase peak
2c	TF binding + matched TF motif + DNase peak
3a	TF binding + any motif + DNase peak
3b	TF binding + matched TF motif
4	TF binding + DNase peak
5	TF binding or DNase peak
6	Motif hit

### Effects of AD-associated SNPs on miRNA binding

MiRNAs are small, non-coding regulatory molecules consisting of approximately 21–25 nucleotides. MiRNAs can inhibit mRNA translation or mediate mRNA decay through complementary binding to the mRNA 3′ untranslated region (3′UTR) in most cases [18]. SNPs in the miRNA seed region will influence the miRNA target binding and selection directly [19]. miRNASNP v2.0 (http://bioinfo.life.hust.edu.cn/miRNASNP2/) is a solid resource for miRNA-related SNP studies and can narrow down the candidate SNPs to the most promising ones [20]. Therefore, we predicted the effect of every SNP on miRNA binding using miRNASNP.

### Effects of AD-associated SNPs on protein phosphorylation

Protein phosphorylation is a dynamic process involving the action and regulation of protein kinases (PKs) and protein phosphatases. In the human genome, approximately 70% of nonsynonymous SNPs are potential phosphorylation-related SNPs (phosSNPs) that may affect protein phosphorylation and play ubiquitous roles in rewiring the biological pathways [21]. In the present study, the PhosSNP 1.0 database (http://phossnp.biocuckoo.org/) was used to identify phosSNPs for AD lead SNPs and their proxy SNPs. phosSNPs can be classified into five types: Type I(+)/(−), change of an amino acid with an S/T/Y residue or change of an S/T/Y residue with another amino acid to create a new or remove an original phosphorylation site; Type II(+)/(−), variations to add or remove adjacent phosphorylation sites; Type III(+)/(−), mutations to change PK types of adjacent phosphorylation sites; Type IV(+)/(−), an amino acid substitution among S, T, or Y that could change the PK types in the phosphorylated position; Type V, a variation resulting in a stop codon, which may remove its phosphorylation sites in the protein C-terminus.

### Gene ontology and pathway enrichment analysis of AD-associated genes

Gene Ontology (GO, http://geneontology.org) is a widely adopted source of gene functional annotations, including biological process, molecular function, and cellular component. The Protein ANalysis THrough Evolutionary Relationships (PANTHER, http://www.pantherdb.org) classification system provides PANTHER GO-slim annotations, which includes all inferred annotations from the GO Phylogenetic Annotation project that have passed an additional expert review process beyond the standard GO experimental annotation process [22]. All AD-associated genes were subjected to pathway enrichment analysis in the Search Tool for the Retrieval of Interacting Genes (STRING, v10.0, http://string-db.org). Statistical enrichment tests were executed on gene lists within the STRING namespace, covering Gene Ontology and pathway annotations [23].

### Protein–protein interaction network

To put forward a full description of a protein’s function, knowledge about its specific interaction partners is an important prerequisite. STRING is a database of predicted and known protein interactions that aim to provide a global perspective for as many organisms as feasible [23, 24]. The interactions include direct (physical) and indirect (functional) associations, which are derived from four sources: genomic context, high-throughput experiments, coexpression (conserved), and previous knowledge [25].

The PPI network was constructed using PPI pairs with protein interaction scores > 0.4. The topological properties of the PPI network, such as betweenness and node degree, were analyzed using the CentiScape v2.1 plug-in within the Cytoscape Desktop v3.4.0 [26, 27]. The node degree is the number of nodes adjacent to a given node. The degree allows for immediate evaluation of the regulatory relevance of the node. In signaling networks, for instance, proteins with high node degrees interact with several other signaling proteins, suggesting a central regulatory role. The betweenness is a node centrality index and is similar to the stress but provides a highly elaborated and informative centrality index. The manner of calculating is by considering couples of nodes (v1, v2) and counting the number of shortest paths that link v1 and v2 and pass through a node n. The value is related to the total number of shortest paths that link v1 and v2. Thus, a node can be traversed by only one path that links v1 and v2, but if this path is the only one connecting v1 and v2, the node n will score a higher betweenness value (in the stress computation would have had a low score). The betweenness of a node in a biological network can indicate the relevance of a protein as functionally capable of holding together communicating proteins. To select functional modules, a functional module analysis of the network was performed by way of CytoCluster plug-in in Cytoscape, with a score > 2.

Hub proteins (genes) have higher node degrees and account for 20% of the total number of nodes. Bottleneck proteins are defined as proteins (genes) that have high degrees of betweenness and account for 20% of the total number of nodes and could connect or act as bridges between subnetworks. Hub genes and bottleneck genes play key roles in the stability.

## Results

### AD-associated SNPs and genes detected by GWASs

A total of 195 GWAS SNPs and 126 genes are associated with AD, with a significant threshold of 5 × 10^−8^ according to NCBI association results. The number of SNPs extends to 533 through searching for proxy SNPs that are in strong LD (*r*^2^ ≥ 0.8) with lead SNPs for AD. Among these SNPs, 366 were mapped to intronic regions, 29 located between genes (intergenic), 11 in 3′UTR, 34 in downstream, 93 in upstream, 6 missense (rs2228145, rs2296160, rs2228467, rs75932628, rs679620, rs429358), and 11 synonymous variants. Detailed information of lead SNPs and proxy SNPs for AD is presented in S1 Table.

### Functional annotation of AD-associated SNPs

Of the 533 AD-associated SNPs examined for possible regulatory functions, 139 had a score of “7”, which means no data were available (or error) for these SNPs in RegulomeDB, and the remaining 394 SNPs returned with scores of 1–6 (S1 Table). A RegulomeDB score < 3 indicated that SNPs had a relatively high degree of evidence for potential regulatory function (‘‘likely to affect binding”). Twenty-six SNPs had a RegulomeDB score of “1”, and 20 returned a RegulomeDB score of “2”. For 3DSNP, 12 SNPs had scores of > 100, and 16 were in the range of 60–100 (S1 Table). Remarkably, the highest evidence of regulatory function was located in the *APOE*-*TOMM40* region. The lead SNP *APOE*/rs439401 had a RegulomeDB score of 1b and 3DSNP score of 152.3. The lead *TOMM40*/SNP rs157580 had a RegulomeDB score of 1f and 3DSNP score of 108.56. RegulomeDB revealed that rs439401 affects the binding of 20 different proteins, including POLR2A, TAL1, MYBL2, SAP30, UBTF, HDAC1, STAT3, ZNF263, GATA1, BRCA1, CTCF, BHLHE40, GATA2, MAX, MYC, REST, TEAD4, USF1, EGR1, and JUNB, and falls within VDR binding motifs.

### Effects of AD-associated SNPs on miRNA binding

Effects of the lead SNPs and their proxy SNPs for AD on miRNA binding were analyzed using miRNASNP (v2). Six lead SNPs and two proxy SNPs that potentially influence the recognition and targeting of miRNA were identified (Table 2).

**Table 2:** SNPs predicted to potentially affect miRNA binding.

SNPs	Gene	SNP location	miRNA(loss)	miRNA(gain)
*GWAS SNPs*				
rs10119(G/A)	*TOMM40*	chr19:44903416		hsa-miR-6128
rs1048699(C/T)	*PPP1R37*	chr19:45147128	hsa-miR-214-3p hsa-miR-761	
rs148763909(C/T)	*SAP30L*	chr5:154457546		hsa-miR-6084 hsa-miR-3620-3p
rs610932(T/G)	*MS4A6A*	chr11:60171834		hsa-miR-626
rs6857(C/T)	*PVRL2*	chr19:44888997		hsa-miR-320e
rs714948 (C/A)	*PVR*	chr19:44662645		hsa-miR-432-5p hsa-miR-3198 hsa-miR-4309
*Proxy SNP*				
rs12539172(T/C)	*NYAP1*	chr7:100091794		hsa-let-7g-3p
rs2847655 (T/C)	*MS4A2*	chr11:60098198		hsa-miR-585

### Effects of AD-associated SNPs on protein phosphorylation

One lead SNP and two proxy SNPs were identified as phosSNPs. Lead SNP rs2296160 located in *CR1* was classified into Type I(−) and Type III(+)/(−). The proxy SNP rs679620, mapped to the *MMP3* gene, was classified into Type II(+) and Type III(+)/(−). The proxy SNP rs2228145, located at *IL6R*, was classified into Type III(+)/(−).

### GO and pathway enrichment analyses of GWAS AD-associated genes

**Fig 2:**
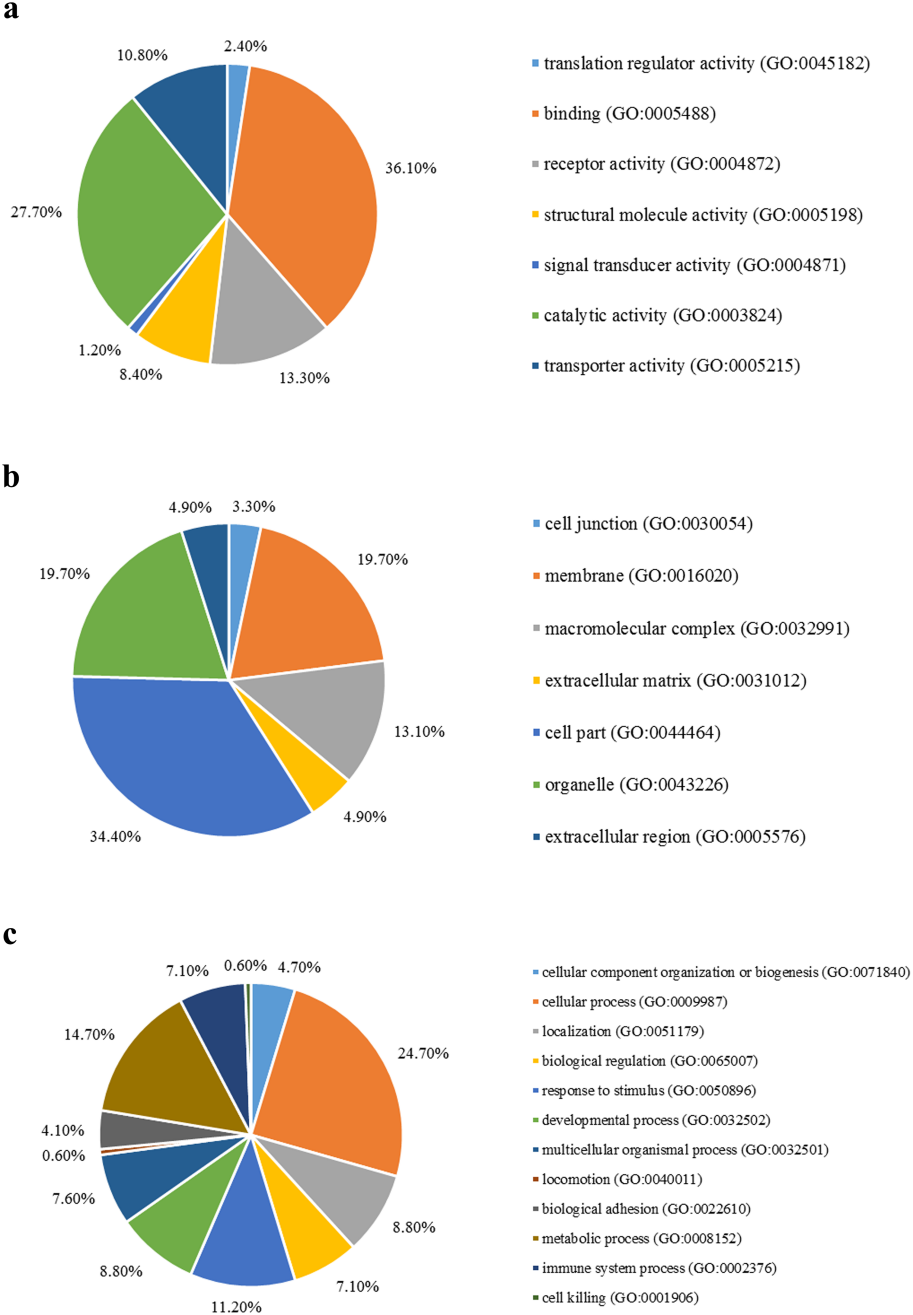
(a) The molecular function, (b) cellular component and (c) biological process of Alzheimer’s disease-associated genes identified by genome-wide association studies.

We first mapped the AD-associated genes onto GO databases via PANTHER using three primary categories: molecular function, cellular component, and biological process (Fig 2). AD-associated genes were mainly enriched in binding function, cell part, and cellular process.

Pathway enrichment analysis of AD-associated genes using STRING uncovered some evidence of over-representation of “regulation of beta-amyloid formation,” “regulation of neurofibrillary tangle assembly,” “leukocyte-mediated immunity,” and “protein–lipid complex assembly” signaling pathway (Table 3).

**Table 3:** Significantly enriched pathways of Alzheimer’s disease-associated genes.

Pathway ID	Pathway description	Observed gene count	False discovery rate	Matching proteins in your network (labels)
GO.1902003	regulation of beta-amyloid formation	5	8.86E-08	*ABCA7*,*APOE*,*CLU*,*PICALM*,*SORL1*
GO.1902996	regulation of neurofibrillary tangle assembly	3	2.61E-05	*APOE*,*CLU*,*SORL1*
GO.0002443	leukocyte mediated immunity	7	6.26E-05	*ACE*,*BCL3*,*CLU*,*CR1*,*HLA-DRB1*,*IL6R*,*INPP5D*
GO.0065005	protein-lipid complex assembly	4	1.59E-04	*ABCA7*,*APOC1*,*APOE*,*BIN1*

### Protein–protein interaction network

**Fig 3:**
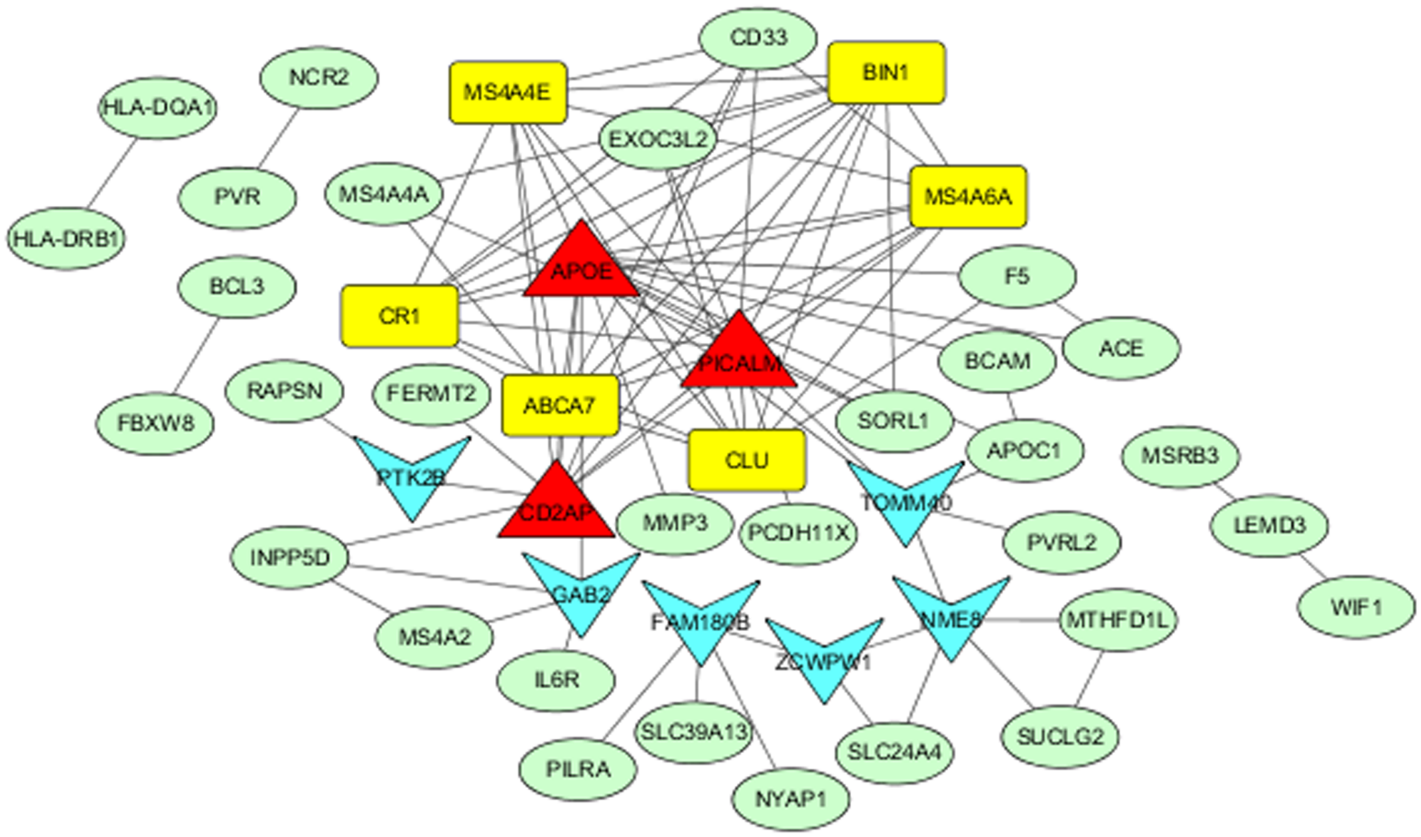
Protein–protein interaction network of Alzheimer’s disease-associated genes. The nodes and edges represent the proteins (genes) and their interactions, respectively. Yellow nodes represent the hub genes, blue nodes represent bottleneck genes, and red nodes represent both types of genes.

**Fig 4:**
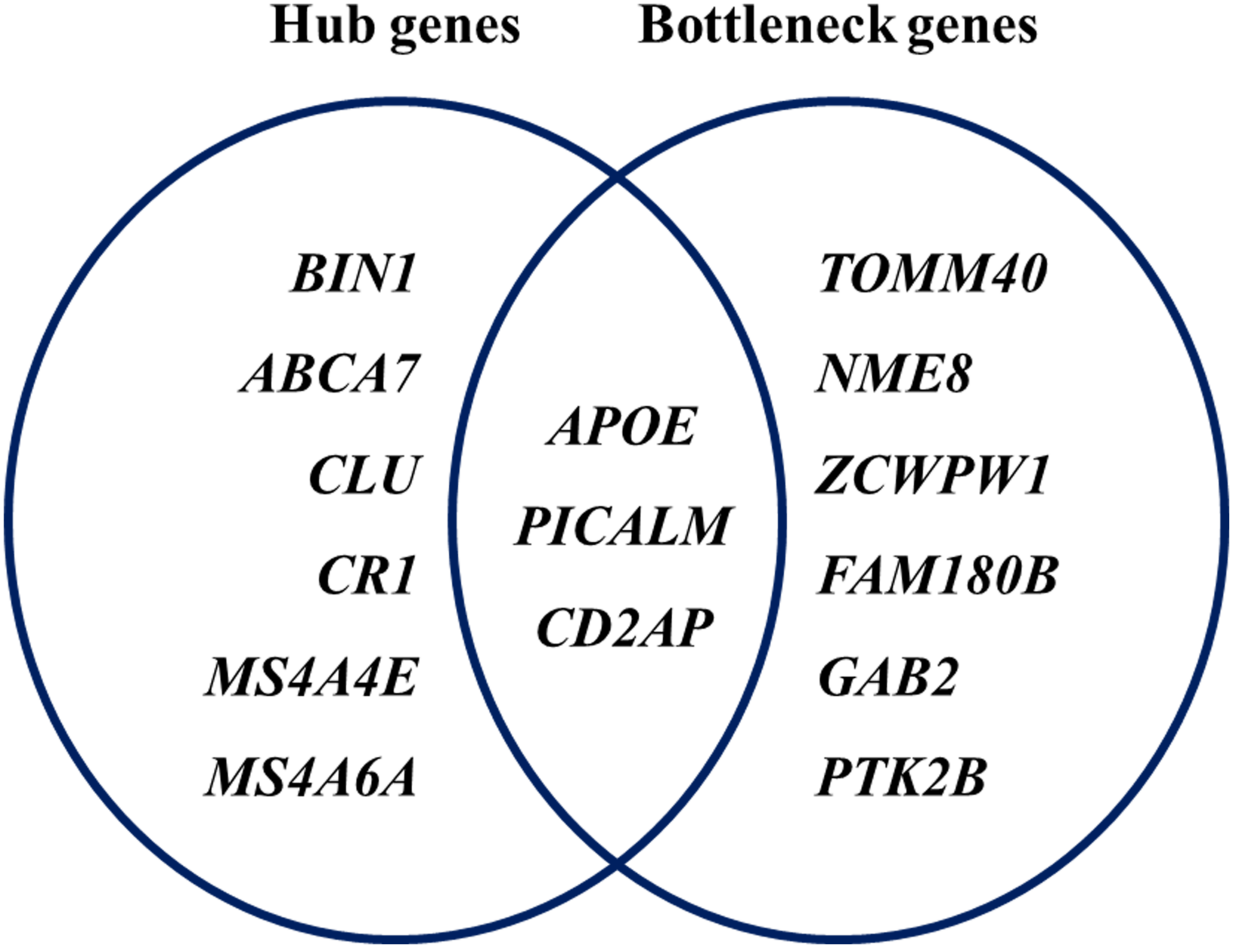
Hub and bottleneck genes in the protein–protein interaction network.

PPI network analysis of 126 AD-associated genes showed significant connectivity among proteins using STRING (v10.0) with default settings (observed interaction, 99; expected interaction, 3.58e + 1; P-value, 0; proteins, 103). The significant PPI pairs with a combined score > 0.4 were selected for constructing the PPI network using Cytoscape. The PPI network contained 47 nodes and 176 edges (Fig 3). The maximum, mean, and minimum of node degree were 16, 3.826, and 1, respectively. In all, the nine hub genes with strong connections were *APOE*, *PICALM*, *BIN1*, *ABCA7*, *CD2AP*, *CLU*, *CR1*, *MS4A4E*, and *MS4A6A* (Table 4). The maximum, mean, and minimum betweenness values were 557.48, 63.78, and 0.00, respectively. The bottleneck genes in the PPI network were *APOE*, *TOMM40*, *NME8*, *PICALM*, *CD2AP*, *ZCWPW1*, *FAM180B*, *GAB2*, and *PTK2B*. Moreover, *APOE*, *PICALM*, and *CD2AP* were both bottleneck genes and hub genes (Fig 4).

**Table 4:** Hub genes associated with Alzheimer’s disease and their interactions.

Node1	Node degree	Node 2
*APOE*	16	*ABCA7*, *ACE*, *APOC1*, *BCAM*, *BIN1*, *CD2AP*, *CLU*, *CR1*, *F5*, *GAB2*, *MMP3*, *MS4A4E*, *MS4A6A*, *PICALM*, *SORL1*, *TOMM40*
*PICALM*	14	*ABCA7*, *APOE*, *BIN1*, *CD2AP*, *CD33*, *CLU*, *CR1*, *EXOC3L2*, *MS4A4A*, *MS4A4E*, *MS4A6A*, *PCDH11X*, *SORL1*, *TOMM40*
*BIN1*	11	*ABCA7*, *APOE*, *CD2AP*, *CLU*, *CR1*, *EXOC3L2*, *MS4A4A*, *MS4A4E*, *MS4A6A*, *PICALM*, *SORL1*
*ABCA7*	10	*APOE*, *BIN1*, *CD2AP*, *CD33*, *CLU*, *CR1*, *MS4A4A*, *MS4A4E*, *MS4A6A*, *PICALM*
*CD2AP*	10	*ABCA7*, *APOE*, *BIN1*, *CD33*, *FERMT2*, *INPP5D*, *MS4A4E*, *MS4A6A*, *PICALM*, *PTK2B*
*CLU*	9	*ABCA7*, *APOE*, *BIN1*, *CR1*, *EXOC3L2*, *F5*, *MS4A4E*, *MS4A6A*, *PICALM*
*CR1*	9	*ABCA7*, *APOE*, *BIN1*, *CD33*, *CLU*, *EXOC3L3*, *MS4A4E*, *MS4A6A*, *PICALM*
*MS4A4E*	9	*ABCA7*, *APOE*, *BIN1*, *CD2AP*, *CD33*, *CLU*, *CR1*, *MS4A6A*, *PICALM*
*MS4A6A*	9	*ABCA7*, *APOE*, *BIN1*, *CD2AP*, *CD33*, *CLU*, *CR1*, *MS4A4E*, *PICALM*

**Fig 5:**
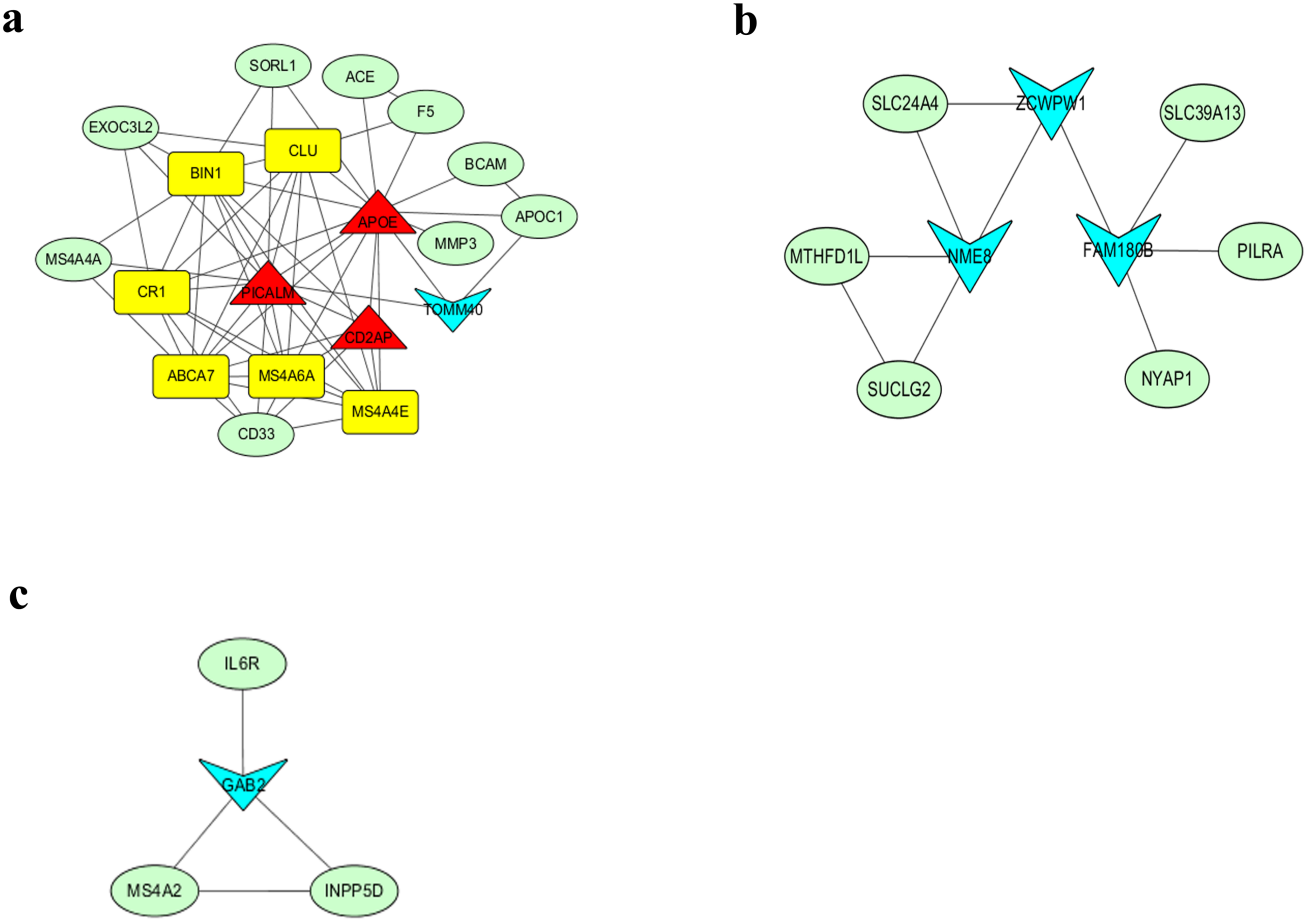
Modules in the protein–protein interaction network. There are 19, 9 and 4 nodes in Modules 1 (a), 2 (b) and 3 (c), respectively. Yellow nodes represent hub genes, blue nodes represent bottleneck genes, green nodes represent neither hub nor bottleneck genes, and red nodes represent both types of genes.

Clustering analysis of the PPI network was performed using CytoCluster in Cytoscape to select functional modules. Three modules with scores of > 2 were identified (Fig 5). Module 1 included 19 genes (*APOE*, *PICALM*, *CD2AP*, *BIN1*, *ABCA7*, *CLU*, *CR1*, *MS4A4E*, *MS4A6A*, *TOMM40*, *CD33*, *APOC1*, *MMP3*, *SORL1*, *MS4A4A*, *EXOC3L2*, *ACE*, *F5*, and *BCAM*). Module 2 included nine genes (*ZCWPW1*, *NME8*, *FAM180B*, *SLC24A4*, *MTHFD1L*, *SUCLG2*, *SLC39A13*, *PLRA*, and *NYAP1*). Module 3 included four genes (*GAB2*, *MS4A2*, *INPP5D*, and *IL6R*) (Fig 5).

## Discussion

To explore functional mechanisms of AD-associated SNPs and genes, we characterized the influence of these SNPs on miRNA binding and protein phosphorylation and conducted GO and pathway enrichment analyses and PPI analysis of AD-associated genes using computational approaches. Our computational analysis identified six lead SNPs (rs10119, rs1048699, rs148763909, rs610932, rs6857, and rs714948) and two proxy SNPs (rs12539172 and rs2847655) that potentially affected miRNA binding. The rs6857 T allele in the 3′UTR of *PVRL2* was predicted to create a binding site for miR-320e. *PVRL2* and miR-320e were expressed in the brain [28]. Moreover, miR-320e decreased the expression level of *PVRL2* and the T allele was associated with a lower expression level of *PVRL2* [29], suggesting that rs6857 increases the risk of AD, at least in part, by downregulating *PVRL2* expression through miR-320e. Furthermore, SNPs rs10119, rs610932, and rs2847655 were correlated with differential expression levels of their host genes in the blood [30]. The effects of rs610932 and rs714948 on the expression levels of *MS4A6A* and *PVR* in the cerebellum and temporal cortex have been reported [31]. Delay et al. [32] recently demonstrated that AD-associated rs7143400-T and rs9909-C alleles regulate *FERMT2* and *NUP160* expressions through miR-4504 and miR-1185-1-3p, respectively. Together, these results showed an action mechanism of SNP on AD by affecting microRNA binding.

Abnormal regulation of protein phosphorylation is known to be related to the pathogenesis of various diseases. Neurofibrillary tangles in AD patients are composed of abnormally phosphorylated tau proteins. Niu et al. [33] reported the associations of *IDUA* phosSNPs rs3755955, rs6831280, and *WNT16* rs2707466 with bone mineral density in GWAS meta-analyses. Deng et al. [34] demonstrated that phosSNP rs6265 influences hip bone mineral density by regulating BDNF protein phosphorylation and osteoblast differentiation. In the present study, we identified one lead SNP, rs2296160, and two proxy SNPs, rs679620 and rs2228145, that may affect protein phosphorylation status, thereby suggesting a similar mechanism for AD.

The present study identified highly interconnected network “hub” genes and bottleneck genes. As expected, *APOE* was the highest ranked AD gene. *APOE* encodes a pleiotropic glycoprotein and has been associated with neuronal repair, nerve generation, activation of lipolytic enzymes, and immune response. The binding of APOE to hydrophobic Aβ peptide leads to synaptic dysfunction and neurodegeneration [35]. Notably, the lead SNP *APOE*/rs439401 had a RegulomeDB score of 1b and 3DSNP score of 152.3. Furthermore, rs439401 is located in the promoter of the *APOC1* gene and affects the binding of 20 different proteins. APOC1 is related to neuronal plasticity through the redistribution of lipids to axons and regeneration of Schwann cells [36]. Moreover, the apoC1 levels are elevated in the hippocampus of Alzheimer sufferers, and apoC1 is assumed to interact with apoE in the lipid metabolism at the cerebral level [37]. GO and pathway enrichment analyses suggested that AD pathogenesis is involved in beta-amyloid formation, neurofibrillary tangle assembly, and immune response. Three hub genes (*CLU*, *CR1*, *ABCA7*) have putative functions in the immune system. A gene-regulatory network study from 1647 AD and control brain samples demonstrated that networks involved in immune specific modules are disrupted in brains with AD [38]. Furthermore, immune response genes and regulatory regions are upregulated in the hippocampus of an inducible mouse model of AD-like neurodegeneration [39]. Our data confirmed previous findings that highlight the importance of the innate immune system in the AD pathophysiology [40]. Three genes (*PICALM*, *CD2AP*, *BIN1*) are involved in the processes of endocytosis. Endocytosis is critical for the normal processing of APP central to AD pathogenesis. Our results are in good agreement with well-known AD pathogenesis [41].

In conclusion, the computational characterization of GWAS AD-associated genes highlighted genes (proteins) and pathways are crucial to AD pathophysiology, thereby enhancing our understanding of this condition and providing potential targets of treatment. Our results revealed the potential functional mechanisms of lead SNPs for AD by influencing miRNA binding and protein phosphorylation, which may need further experimental tests in the future.

## Supporting information

S1 Table Information of SNPs and genes for Alzheimer disease revealed by GWASs. (DOCX)
