# Supplementary material for: Functional annotation of Alzheimer's disease associated loci revealed by GWASs
Source: PLoS One. 2017 Jun 26;12(6):e0179677. doi: 10.1371/journal.pone.0179677 (PMC5484478; doi:10.1371/journal.pone.0179677)
Supplement: S1 Table — (DOCX) [file pone.0179677.s001.docx]

Table S1

| **Locus** | **SNPs** | **Ref** **Alt** | | **Risk allele** | **Gene** | **Position** | **Coordinate(0-based)** | | **Regulome DB Score** | **3DSNP Score** |
| --- | --- | --- | --- | --- | --- | --- | --- | --- | --- | --- |
| **1q21** | rs6684439 | C | T | No Data | *IL6R* | intron variant | chr01 | 154395838 | 5 | 64.15 |
|  | rs7518199 | A | C | No Data | *IL6R* | intron variant | chr01 | 154407418 | 5 | 26 |
|  | rs4845622 | A | C | No Data | *IL6R* | intron variant | chr01 | 154411418 | 5 | 13.73 |
|  | rs4393147 | C | T | No Data | *IL6R* | intron variant | chr01 | 154414036 | 3a | 27.27 |
|  | rs4453032 | A | G | No Data | *IL6R* | intron variant | chr01 | 154414085 | 4 | 28.23 |
|  | rs6664201 | C | T | No Data | *IL6R* | intron variant | chr01 | 154414295 | 5 | 41.4 |
|  | rs4845372 | C | A | No Data | *IL6R* | intron variant | chr01 | 154415395 | 5 | 97.07 |
|  | rs4845623 | A | G | No Data | *IL6R* | intron variant | chr01 | 154415776 | 5 | 22.9 |
|  | rs12730036 | C | T | No Data | *IL6R* | intron variant | chr01 | 154416968 | 5 | 12.87 |
|  | rs4845373 | C | T | No Data | *IL6R* | intron variant | chr01 | 154417828 | 4 | 36.02 |
|  | rs11265613 | T | C | No Data | *IL6R* | intron variant | chr01 | 154418414 | 6 | 17.67 |
|  | rs4576655 | C | T | No Data | *IL6R* | intron variant | chr01 | 154418748 | 5 | 24.77 |
|  | rs4537545 | C | T | No Data | *IL6R* | intron variant | chr01 | 154418878 | 4 | 24.4 |
|  | **rs61812598** | G | A | G | *IL6R* | intron variant | chr01 | 154420086 | No Data | 7.03 |
|  | rs7529229 | T | C | No Data | *IL6R* | intron variant | chr01 | 154420777 | 2b | 9.21 |
|  | rs4129267 | C | T | No Data | *IL6R* | intron variant | chr01 | 154426263 | 2b | 58.33 |
|  | rs2228145 | A | C | No Data | *IL6R* | intron variant, missense | chr01 | 154426969 | 5 | 10.89 |
| **1q23** | **rs6703865** | G | A | A | *F5* | intron variant | chr01 | 169550962 | 6 | 1.76 |
| **1q32** | **rs6656401** | A | G | A | *CR1* | intron variant | chr01 | 207692048 | 5 | 1.49 |
|  | **rs679515** | T | C | T | *CR1* | intron variant | chr01 | 207750567 | 6 | 0.86 |
|  | **rs3818361** | A | G | ? | *CR1* | intron variant | chr01 | 207784967 | 5 | 1.24 |
|  | **rs6701713** | A | G | A | *CR1* | intron variant | chr01 | 207786288 | 4 | 4.41 |
|  | **rs2296160** | A | G | No Data | *CR1* | missense | chr01 | 207795319 | No Data | 0.86 |
|  | **rs1408078** | T | C | No Data | *CR1* | intron variant | chr01 | 207800554 | 5 | 1.21 |
|  | **rs4844610** | A | C | No Data | *CR1* | intron variant | chr01 | 207802551 | 5 | 2.74 |
|  | **rs1408077** | A | C | No Data | *CR1* | intron variant | chr01 | 207804140 | 6 | 2.98 |
| **2p12** | **rs2298948** | T | C | C | *GCFC2* | intron variant | chr02 | 75926564 | 5 | 1.28 |
|  | **rs6738962** | A | G | ? | *CTNNA2* | intron variant | chr02 | 80281172 | 3a | 6.15 |
| **2q13** | **rs4676049** | C | T | A | *EDAR;LOC105373544* | intergenic | chr02 | 109635256 | 3a | 1.94 |
| **2q14** | **rs12989701** | C | A | ? | *BIN1* | intron variant | chr02 | 127887984 | 3a | 14.95 |
|  | **rs11680911** | A | C | No Data | *BIN1* | intron variant | chr02 | 127888756 | 5 | 1.04 |
|  | **rs7561528** | G | A | A | *BIN1* | intron variant | chr02 | 127889636 | 6 | 1.58 |
|  | **rs4663105** | A | C | C | *BIN1* | intron variant | chr02 | 127891426 | 5 | 2.26 |
|  | **rs6733839** | C | T | T | *BIN1* | intron variant | chr02 | 127892809 | 5 | 6.16 |
|  | **rs744373** | A | G | ? | *BIN1;CYP27C1* | downstream | chr02 | 127894614 | 5 | 5.66 |
| **2q37** | **rs35349669** | C | T | T | *INPP5D* | intron variant | chr02 | 234068475 | 5 | 6.28 |
|  | rs28669088 | C | T | No Data | *INPP5D* | intron variant | chr02 | 234068704 | No Data | 3.39 |
|  | rs28534487 | C | A | No Data | *INPP5D* | intron variant | chr02 | 234069265 | 5 | 5.26 |
|  | rs28655385 | C | A | No Data | *INPP5D* | intron variant | chr02 | 234069322 | 5 | 3.36 |
|  | rs28459768 | A | C | No Data | *INPP5D* | intron variant | chr02 | 234069511 | 6 | 3.39 |
|  | rs28478933 | C | A | No Data | *INPP5D* | intron variant | chr02 | 234069763 | No Data | 3.3 |
|  | rs28539971 | G | A | No Data | *INPP5D* | intron variant | chr02 | 234070041 | 6 | 3.36 |
|  | rs35877172 | C | T | No Data | *INPP5D* | intron variant | chr02 | 234070084 | 5 | 3.39 |
|  | rs28605534 | G | C | No Data | *INPP5D* | intron variant | chr02 | 234070170 | 5 | 3.69 |
|  | rs28609111 | G | A | No Data | *INPP5D* | intron variant | chr02 | 234070189 | 5 | 3.32 |
|  | rs28576692 | T | C | No Data | *INPP5D* | intron variant | chr02 | 234070232 | No Data | 4 |
|  | rs7607812 | G | A | No Data | *INPP5D* | intron variant | chr02 | 234071141 | No Data | 4.53 |
|  | rs55801407 | G | A | No Data | *INPP5D* | intron variant | chr02 | 234071161 | 6 | 3.38 |
|  | rs7568027 | A | G | No Data | *INPP5D* | intron variant | chr02 | 234071240 | 5 | 3.65 |
|  | rs7559212 | A | C | No Data | *INPP5D* | intron variant | chr02 | 234071248 | 5 | 4.24 |
|  | rs7607736 | C | T | No Data | *INPP5D* | intron variant | chr02 | 234074300 | 4 | 31.68 |
| **3p21** | **rs2228467** | T | C | G | *ACKR2* | missense | chr03 | 42906115 | 5 | 5.27 |
|  | **rs6808835** | G | T | G | *CCRL2;LOC102724297* | synonymous codon,  upstream variant 2KB | chr03 | 46449863 | 5 | 41.11 |
| **3p14** | **rs62256378** | G | A | A | *SUCLG2* | intron variant | chr03 | 67457032 | No Data | 1.66 |
|  | rs74727963 | C | T | No Data | *SUCLG2* | intron variant | chr03 | 67457519 | 6 | 1.64 |
|  | rs78609144 | C | T | No Data | *SUCLG2* | intron variant | chr03 | 67459075 | No Data | 2.29 |
|  | rs79963093 | G | A | No Data | *SUCLG2* | intron variant | chr03 | 67460765 | No Data | 1.39 |
|  | rs80028595 | T | C | No Data | *SUCLG2* | intron variant | chr03 | 67463214 | 6 | 1.97 |
|  | rs77072359 | C | T | No Data | *SUCLG2* | intron variant | chr03 | 67466389 | 6 | 5.85 |
|  | rs74680427 | C | T | No Data | *SUCLG2* | intron variant | chr03 | 67492299 | 6 | 2.59 |
| **3q26** | **rs509208** |  |  | G | *LOC100420620;RN7SKP298* | intergenic | chr03 | 166014468 | 5 | 1.34 |
| **3q28** | **rs9877502** | G | A | A | *EMNC;OSTN* | upstream | chr03 | 190669517 | 6 | 1.27 |
| **5q11** | **rs4700060** | C | T | ? | *ANKRD55* | intron variant | chr05 | 55510655 | No Data | 2.65 |
| **5q14** | rs304132 | A | G | No Data | *MEF2C-AS1* | intron variant | chr05 | 88215593 | 5 | 1.47 |
|  | **rs190982** | G | A | A | *MEF2C-AS1* | intron variant | chr05 | 88223419 | No Data | 1.51 |
| **5q21.3** | **rs112724034** | C | T | ? | *LOC100289673* | nc transcript variant | chr05 | 109221025 | 5 | 9.82 |
| **5q33** | **rs148763909** | C | T | ? | *SAP30L* | nc transcript variant,  utr variant 3 prime | chr05 | 153837105 | 4 | 4.35 |
| **6p21** | **rs9271192** | C | A | C | *HLA-DRB1;HLA-DQA1* | upstream | chr06 | 32578529 | 5 | 5.55 |
|  | rs77579690 | G | A | No Data | *LOC105375054* | nc transcript | chr06 | 40826389 | 6 | 1.89 |
|  | rs12664332 | G | A | No Data | *LOC101929555* | intron variant | chr06 | 40904029 | 5 | 13.42 |
|  | **rs75932628** | C | T | T | *LOC105375056;TREM2* | intron variant, missense | chr06 | 41129251 | 5 | 11.16 |
|  | **rs6922617** | G | A | A | *NCR2;FOXP4-AS1* | downstream | chr06 | 41336100 | No Data | 2.07 |
|  | **rs11966476** | G | A | No Data | *NCR2;FOXP4-AS1* | downstream | chr06 | 41340571 | 4 | 107.23 |
| **6p12** | rs1931833 | T | A | No Data | *TNFRSF21;CD2AP* | upstream | chr06 | 47427280 | No Data | 5.88 |
|  | rs7740963 | C | T | No Data | *TNFRSF21;CD2AP* | upstream | chr06 | 47427610 | No Data | 11.44 |
|  | rs9381562 | C | A | No Data | *TNFRSF21;CD2AP* | upstream | chr06 | 47429766 | 5 | 2.46 |
|  | rs9473117 | A | C | No Data | *TNFRSF21;CD2AP* | upstream | chr06 | 47431283 | No Data | 1.21 |
|  | rs9369693 | A | C | No Data | *TNFRSF21;CD2AP* | upstream | chr06 | 47433750 | 6 | 2.05 |
|  | rs9369695 | C | A | No Data | *TNFRSF21;CD2AP* | upstream | chr06 | 47440564 | 6 | 2.32 |
|  | rs4715018 | T | C | No Data | *TNFRSF21;CD2AP* | upstream | chr06 | 47441870 | 4 | 3.61 |
|  | rs1931837 | C | T | No Data | *TNFRSF21;CD2AP* | upstream | chr06 | 47442376 | 5 | 1.77 |
|  | rs9381564 | A | G | No Data | *CD2AP* | upstream variant 2KB | chr06 | 47443805 | 5 | 14.73 |
|  | rs1004173 | C | T | No Data | *CD2AP* | upstream variant 2KB | chr06 | 47445016 | 2b | 143.49 |
|  | rs4715019 | T | A | No Data | *CD2AP* | intron variant | chr06 | 47447040 | 5 | 101.58 |
|  | rs9367279 | A | G | No Data | *CD2AP* | intron variant | chr06 | 47448335 | 2b | 46.75 |
|  | rs9473119 | G | A | No Data | *CD2AP* | intron variant | chr06 | 47450617 | 6 | 16.18 |
|  | rs9296558 | C | T | No Data | *CD2AP* | intron variant | chr06 | 47451882 | 4 | 46.26 |
|  | rs9296559 | T | C | No Data | *CD2AP* | intron variant | chr06 | 47452269 | 3a | 38.69 |
|  | **rs9349407** | G | C | C | *CD2AP* | intron variant | chr06 | 47453377 | 4 | 11.09 |
|  | rs4711878 | A | G | No Data | *CD2AP* | intron variant | chr06 | 47456117 | 6 | 1.38 |
|  | rs10948361 |  |  | No Data | *CD2AP* | intron variant | chr06 | 47457708 | 3a | 2.66 |
|  | rs6931478 | C | A | No Data | *CD2AP* | intron variant | chr06 | 47461912 | 5 | 9.41 |
|  | rs9349409 | G | T | No Data | *CD2AP* | intron variant | chr06 | 47463125 | 3a | 12.15 |
|  | rs9395262 | T | C | No Data | *CD2AP* | intron variant | chr06 | 47465266 | 5 | 5.87 |
|  | rs7738044 | A | G | No Data | *CD2AP* | intron variant | chr06 | 47469272 | 5 | 25.72 |
|  | rs9473122 | C | T | No Data | *CD2AP* | intron variant | chr06 | 47474961 | 5 | 1.58 |
|  | rs9473123 | G | A | No Data | *CD2AP* | intron variant | chr06 | 47475338 | 6 | 2.69 |
|  | rs9463335 | G | A | No Data | *CD2AP* | intron variant | chr06 | 47479135 | No Data | 2.17 |
|  | rs13211285 | C | T | No Data | *CD2AP* | intron variant | chr06 | 47479617 | No Data | 3.46 |
|  | rs4711880 | A | G | No Data | *CD2AP* | intron variant | chr06 | 47480675 | 3a | 4.71 |
|  | rs1872505 | C | G | No Data | *CD2AP* | intron variant | chr06 | 47480974 | 5 | 3.62 |
|  | rs9473126 | C | G | No Data | *CD2AP* | intron variant | chr06 | 47481832 | No Data | 1.53 |
|  | rs4715025 | C | G | No Data | *CD2AP* | intron variant | chr06 | 47483652 | No Data | 2.19 |
|  | rs7749271 | T | C | No Data | *CD2AP* | intron variant | chr06 | 47485001 | No Data | 1.61 |
|  | rs7767350 | C | T | No Data | *CD2AP* | intron variant | chr06 | 47485125 | No Data | 1.25 |
|  | **rs10948363** | A | G | G | *CD2AP* | intron variant | chr06 | 47487761 | 6 | 5.56 |
|  | rs9296561 | G | A | No Data | *CD2AP* | intron variant,  upstream variant 2KB | chr06 | 47488937 | No Data | 1.7 |
|  | rs7749167 | G | A | No Data | *CD2AP* | intron variant | chr06 | 47493939 | 5 | 2.68 |
|  | rs9296564 | A | G | No Data | *CD2AP* | intron variant | chr06 | 47494758 | 6 | 1.68 |
|  | rs7754282 | G | C | No Data | *CD2AP* | intron variant | chr06 | 47502023 | 4 | 1.8 |
|  | rs6904764 | A | T | No Data | *CD2AP* | intron variant | chr06 | 47503496 | 4 | 3.68 |
|  | rs9473128 | C | T | No Data | *CD2AP* | intron variant | chr06 | 47505009 | 4 | 1.86 |
|  | rs9349413 | A | G | No Data | *CD2AP* | intron variant | chr06 | 47511490 | 6 | 2.29 |
|  | rs2151974 | G | A | No Data | *CD2AP* | intron variant | chr06 | 47515629 | 6 | 1.53 |
|  | rs2151975 | T | C | No Data | *CD2AP* | intron variant | chr06 | 47515662 | No Data | 1.89 |
|  | rs2171089 | A | G | No Data | *CD2AP* | intron variant | chr06 | 47515810 | 5 | 1.61 |
|  | rs9367284 | A | G | No Data | *CD2AP* | intron variant | chr06 | 47516368 | 6 | 1.25 |
|  | rs9381575 | C | G | No Data | *CD2AP* | intron variant | chr06 | 47528763 | 6 | 1.99 |
|  | rs9395279 | C | T | No Data | *CD2AP* | intron variant | chr06 | 47542863 | 6 | 1.54 |
|  | rs9357546 | C | T | No Data | *CD2AP* | intron variant | chr06 | 47549494 | 3a | 2.49 |
|  | rs9395283 | T | C | No Data | *CD2AP* | intron variant | chr06 | 47551443 | 5 | 1.41 |
|  | rs9349415 | T | C | No Data | *CD2AP* | intron variant | chr06 | 47551860 | 6 | 1.46 |
|  | rs9349416 | G | A | No Data | *CD2AP* | intron variant | chr06 | 47551937 | No Data | 2.11 |
|  | rs9369716 | A | T | No Data | *CD2AP* | intron variant | chr06 | 47552179 | 5 | 1.74 |
|  | rs9296567 | G | A | No Data | *CD2AP* | intron variant | chr06 | 47553401 | 6 | 1.53 |
|  | rs9395285 | G | A | No Data | *CD2AP* | intron variant | chr06 | 47554176 | No Data | 2.14 |
|  | rs9369717 | T | G | No Data | *CD2AP* | intron variant | chr06 | 47554467 | 5 | 1.42 |
|  | rs1485780 | A | C | No Data | *CD2AP* | intron variant | chr06 | 47556629 | No Data | 1.53 |
|  | rs9381578 | C | T | No Data | *CD2AP* | intron variant | chr06 | 47556633 | No Data | 1.14 |
|  | rs9381579 | C | T | No Data | *CD2AP* | intron variant | chr06 | 47556679 | No Data | 1.35 |
|  | rs10456570 | A | G | No Data | *CD2AP* | intron variant | chr06 | 47559450 | No Data | 1.13 |
|  | rs6903331 | A | G | No Data | *CD2AP* | intron variant | chr06 | 47562914 | 6 | 1.66 |
|  | rs2396825 | A | G | No Data | *CD2AP* | intron variant | chr06 | 47568695 | No Data | 1.19 |
|  | rs9395286 | T | C | No Data | *CD2AP* | intron variant | chr06 | 47575331 | 5 | 1.94 |
|  | rs13212790 | T | C | No Data | *CD2AP* | intron variant | chr06 | 47576366 | 6 | 1.82 |
|  | rs9349417 | A | G | No Data | *CD2AP* | intron variant | chr06 | 47580656 | No Data | 1.43 |
|  | rs9381581 | G | A | No Data | *CD2AP* | intron variant | chr06 | 47580694 | No Data | 1.06 |
|  | rs9463342 | A | T | No Data | *CD2AP* | intron variant | chr06 | 47585105 | 6 | 0.9 |
|  | rs10948367 | A | G | No Data | *CD2AP* | intron variant | chr06 | 47585614 | No Data | 2.26 |
|  | rs2171086 | C | T | No Data | *CD2AP* | intron variant | chr06 | 47590103 | No Data | 1.4 |
|  | rs7754971 | C | T | No Data | *CD2AP* | intron variant | chr06 | 47590475 | No Data | 1.05 |
| **6p11** | **rs1936246** | T | G | T | *LOC101927293* | intron variant | chr06 | 58371947 | No Data | 0.93 |
| **6q14** | **rs1925690** | T | C | ? | *ZNF292* | intron variant | chr06 | 87867062 | No Data | 45.45 |
| **6q23-q24** | **rs11154851** | C | T | ? | *LOC644135;PDE7B* | downstream variant 500B,  intron variant,  nc transcript variant | chr06 | 136368004 | 6 | 1.11 |
| **6q25** | **rs11754661** | G | A | A | *MTHFD1L* | intron variant | chr06 | 151207077 | No Data | 2.48 |
| **7p21** | **rs116139393** | C | A | C | *ZNF12;RSPH10B2* | upstream | chr07 | 6771659 | 6 | #N/A |
|  | **rs58370486** | A | G | ? | *BZW2* | intron variant | chr07 | 16707860 | 2a | 145.09 |
| **7p14** | **rs2718058** | A | G | A | *GPR141;NME8* | intergenic | chr07 | 37841533 | No Data | 1.78 |
|  | rs4723711 | T | A | No Data | *GPR141;NME8* | intergenic | chr07 | 37844262 | 5 | 2.47 |
| **7q22** | rs2405442 | T | C | No Data | *PILRA* | synonymous codon | chr07 | 99971312 | 5 | 38.32 |
|  | rs2906657 | T | C | No Data | *PILRA* | intron variant | chr07 | 99984088 | 6 | 17.57 |
|  | rs34995835 | T | G | No Data | *PILRA* | intron variant | chr07 | 99990363 | 6 | 17.99 |
|  | **rs1476679** | C | T | T | *ZCWPW1* | intron variant | chr07 | 100004445 | 1f | 23.19 |
|  | rs34919929 | G | A | No Data | *ZCWPW1* | intron variant | chr07 | 100012333 | 6 | 7.38 |
|  | rs5015756 | T | C | No Data | *ZCWPW1* | intron variant | chr07 | 100013456 | 5 | 7.63 |
|  | rs6971558 | A | T | No Data | *NYAP1* | upstream variant 2KB | chr07 | 100079856 | No Data | 16.15 |
|  | rs12539172 | T | C | No Data | *NYAP1* | utr variant 3 prime | chr07 | 100091794 | 4 | 93.54 |
| **7q32** | **rs277470** | C | G | No Data | *LOC105375510;*  *PLXNA4* | intron variant | chr07 | 132110922 | 5 | 16.04 |
| **7q35** | **rs11767557** | T | C | ? | *EPHA1-AS1* | intron variant | chr07 | 143109138 | 5 | 24.78 |
|  | **rs11771145** | G | A | G | *EPHA1-AS1* | intron variant | chr07 | 143110761 | 5 | 9.47 |
| **8p21** | **rs28834970** | T | C | C | *PTK2B* | intron variant | chr08 | 27195120 | 5 | 8.39 |
|  | rs6987305 | G | A | No Data | *PTK2B* | intron variant | chr08 | 27208125 | No Data | 25.36 |
|  | rs2322599 | G | A | No Data | *PTK2B* | intron variant | chr08 | 27211909 | 5 | 25.2 |
|  | rs73223431 | C | T | No Data | *PTK2B* | intron variant | chr08 | 27219986 | 2b | 162.31 |
|  | rs17057043 | G | A | No Data | *PTK2B* | intron variant | chr08 | 27220309 | 1d | 60.52 |
|  | rs755951 | A | C | No Data | *PTK2B* | intron variant | chr08 | 27226789 | 3a | 31.23 |
|  | **rs2279590** | T | C | ? | *CLU* | intron variant | chr08 | 27456252 | 3a | 6.71 |
|  | rs7982 | A | G | No Data | *CLU* | nc transcript variant,  synonymous codon | chr08 | 27462480 | 5 | 1.89 |
|  | **rs11136000** | T | C | ? | *CLU* | intron variant | chr08 | 27464518 | 6 | 4.68 |
|  | rs4236673 | A | G | No Data | *CLU* | intron variant | chr08 | 27464928 | 6 | 3.47 |
|  | rs11787077 | T | C | No Data | *CLU* | intron variant | chr08 | 27465311 | 5 | 12.1 |
|  | rs1532276 | T | C | No Data | *CLU* | intron variant | chr08 | 27466156 | 4 | 51.03 |
|  | rs1532277 | T | C | No Data | *CLU* | intron variant | chr08 | 27466180 | 4 | 48.86 |
|  | rs1532278 | T | C | ? | *CLU* | intron variant | chr08 | 27466314 | 2b | 63.71 |
|  | **rs9331896** | C | T | T | *CLU;*  *MIR6843* | downstream variant 500B,  intron variant | chr08 | 27467685 | 5 | 40.02 |
|  | rs2070926 | C | G | No Data | *CLU;*  *MIR6843* | downstream variant 500B,  intron variant | chr08 | 27467820 | 4 | 58.87 |
|  | rs867230 | C | A | No Data | *CLU;*  *MIR6843* | intron variant,  upstream variant 2KB | chr08 | 27468502 | 2b | 87.43 |
|  | **rs9331888** | C | G | No Data | *CLU;*  *MIR6843* | intron variant,  nctranscriptvariant,  upstreamvariant2KB | chr08 | 27468861 | 2b | 81.41 |
|  | **rs569214** | G | T | ? | *CLU;SCARA3* | upstream | chr08 | 27487789 | 5 | 4.43 |
| **9p24** | **rs622536** | T | C | No Data | *GLIS3* | intron variant | chr09 | 3927703 | No Data | 1.23 |
|  | **rs622951** | C | T | No Data | *GLIS3* | intron variant | chr09 | 3927803 | No Data | 1.39 |
|  | **rs623295** | A | C | No Data | *GLIS3* | intron variant | chr09 | 3927840 | 6 | 2.06 |
|  | **rs624290** | C | T | No Data | *GLIS3* | intron variant | chr09 | 3928114 | 5 | 1.01 |
|  | **rs514716** | C | T | G | *GLIS3* | intron variant | chr09 | 3929423 | 5 | 6.13 |
| **10p13** | **rs201119** | T | C | No Data | *CELF2;LOC105376404* | downstreamvariant500B,  intron variant | chr10 | 11049976 | 5 | 8.58 |
|  | **rs7081208** | G | A | A | *FRMD4A* | intron variant | chr10 | 13991864 | 4 | 5.69 |
| **11p15** | **rs11023139** | G | A | ? | *SPON1* | intron variant | chr11 | 14224345 | 4 | 5.45 |
| **11p11** | rs10769256 | C | T | No Data | *SPI1* | intron variant | chr11 | 47378395 | 2b | 14.11 |
|  | rs2293576 | G | A | No Data | *SLC39A13* | intron variant,  nc transcript variant,  synonymous codon | chr11 | 47434985 | 1f | 10.31 |
|  | rs7103835 | G | A | No Data | *RAPSN* | intron variant | chr11 | 47461692 | 1d | 8.99 |
|  | rs11039244 | G | A | No Data | *RAPSN* | intron variant | chr11 | 47466441 | 6 | 5.11 |
|  | rs12361415 | T | G | No Data | *RAPSN;CELF1* | intergenic | chr11 | 47474145 | 6 | 6.8 |
|  | rs12224672 | C | T | No Data | *CELF1* | intron variant | chr11 | 47500399 | 5 | 10.11 |
|  | rs7933019 | G | C | No Data | *CELF1* | intron variant | chr11 | 47509136 | 1f | 1.5 |
|  | rs6485758 | G | A | No Data | *CELF1* | intron variant | chr11 | 47530023 | 1f | 59.82 |
|  | **rs10838725** | T | C | C | *CELF1* | intron variant | chr11 | 47557870 | 6 | 11.19 |
|  | rs61895112 | T | C | No Data | *CELF1* | intron variant | chr11 | 47560432 | 5 | 53.19 |
|  | rs11039284 | A | G | No Data | *CELF1* | intron variant | chr11 | 47564784 | No Data | 13.76 |
|  | rs10838726 | C | G | No Data | *CELF1* | intron variant | chr11 | 47568343 | 6 | 7.5 |
|  | rs11039290 | G | A | No Data | *CELF1* | intron variant | chr11 | 47572278 | 1b | 58.25 |
|  | rs2280231 | C | T | No Data | *KBTBD4;*  *NDUFS3* | intron variant,  upstream variant 2KB  utr variant 5 prime | chr11 | 47600437 | 1d | 201.07 |
|  | rs12287076 | G | C | No Data | *FAM180B* | upstream variant 2KB | chr11 | 47606864 | 4 | 3.25 |
|  | rs10838731 | C | T | No Data | *FAM180B* | upstream variant 2KB | chr11 | 47607134 | 5 | 1.21 |
|  | rs7120548 | T | C | No Data | *MTCH2* | intron variant | chr11 | 47662931 | 1f | 103.17 |
|  | rs11039332 | G | A | No Data | *AGBL2* | intron variant | chr11 | 47695839 | 6 | 4.66 |
|  | rs12365079 | A | T | No Data | *AGBL2* | intron variant | chr11 | 47696595 | 5 | 1.5 |
|  | rs12577383 | C | T | No Data | *FNBP4* | intron variant | chr11 | 47774237 | No Data | 1.88 |
|  | rs7927445 | G | T | No Data | *FNBP4* | intron variant | chr11 | 47780753 | 5 | 3.33 |
|  | rs12223593 | T | G | No Data | *FNBP4* | upstream variant 2KB | chr11 | 47789081 | 4 | 201.72 |
|  | rs7114011 | A | C | No Data | *NUP160* | intron variant | chr11 | 47811308 | 1f | 1.14 |
|  | rs7131262 | T | A | No Data | *NUP160* | intron variant | chr11 | 47836301 | 6 | 1.88 |
|  | rs7934481 | C | T | No Data | *NUP160;PTPRJ* | upstream | chr11 | 47883336 | 4 | 19.65 |
| **11q12** | rs2583476 | G | A | No Data | *MS4A2* | intron variant | chr11 | 59857580 | 5 | 0.85 |
|  | rs2847663 | C | G | No Data | *MS4A2* | intron variant | chr11 | 59858035 | 5 | 1.3 |
|  | rs2847664 | G | A | No Data | *MS4A2* | intron variant | chr11 | 59858496 | No Data | 2.35 |
|  | rs2847666 | A | G | No Data | *MS4A2* | intron variant | chr11 | 59859575 | 6 | 1.06 |
|  | rs2847667 | C | T | No Data | *MS4A2* | intron variant | chr11 | 59859608 | No Data | 0.87 |
|  | rs2583471 | G | A | No Data | *MS4A2* | intron variant | chr11 | 59861813 | No Data | 0.91 |
|  | rs2070970 | C | T | No Data | *MS4A2* | intron variant | chr11 | 59861982 | 6 | 1.09 |
|  | rs2847668 | A | T | No Data | *MS4A2* | intron variant | chr11 | 59862260 | No Data | 1.21 |
|  | rs2847655 | T | C | No Data | *MS4A2* | utr variant 3 prime | chr11 | 59865670 | 6 | 1.85 |
|  | rs2855017 | C | T | No Data | *MS4A2* | downstream variant 500B | chr11 | 59866308 | 3a | 9.69 |
|  | rs17528859 | T | C | No Data | *MS4A2;LOC105369319* | downstream | chr11 | 59867378 | No Data | 1.27 |
|  | rs1813217 | G | C | No Data | *MS4A2;LOC105369319* | downstream | chr11 | 59872497 | 4 | 1.37 |
|  | rs11230147 | C | T | No Data | *MS4A2;LOC105369319* | downstream | chr11 | 59877393 | No Data | 0.91 |
|  | rs4939311 | C | T | No Data | *MS4A2;LOC105369319* | downstream | chr11 | 59877966 | 6 | 1.49 |
|  | rs1125357 | A | C | No Data | *MS4A2;LOC105369319* | downstream | chr11 | 59885492 | 6 | 0.95 |
|  | rs483629 | C | T | No Data | *MS4A2;LOC105369319* | downstream | chr11 | 59899910 | 6 | 0.97 |
|  | rs4939312 | C | T | No Data | *MS4A2;LOC105369319* | downstream | chr11 | 59900105 | No Data | 1.08 |
|  | rs4939314 | T | C | No Data | *MS4A2;LOC105369319* | downstream | chr11 | 59900574 | No Data | 1.34 |
|  | rs61901691 | C | T | No Data | *MS4A2;LOC105369319* | downstream | chr11 | 59902798 | No Data | 1.34 |
|  | rs55847558 | C | G | No Data | *MS4A2;LOC105369319* | downstream | chr11 | 59903792 | No Data | 1.31 |
|  | rs11230155 | C | T | No Data | *MS4A2;LOC105369319* | downstream | chr11 | 59904958 | No Data | 1.31 |
|  | rs2165525 | A | C | No Data | *MS4A2;LOC105369319* | downstream | chr11 | 59906791 | No Data | 1.31 |
|  | rs7124974 | G | T | No Data | *MS4A2;LOC105369319* | downstream | chr11 | 59906971 | 6 | 1.31 |
|  | rs11230160 | C | T | No Data | *MS4A2;LOC105369319* | downstream | chr11 | 59919164 | No Data | 1.34 |
|  | rs11230161 | C | A | No Data | *MS4A2;LOC105369319* | downstream | chr11 | 59920230 | 6 | 1.32 |
|  | rs10897009 | G | A | No Data | *MS4A2;LOC105369319* | downstream | chr11 | 59923193 | No Data | 1.05 |
|  | **rs983392** | A | G | A | *MS4A2;LOC105369319* | downstream | chr11 | 59923507 | 4 | 2.92 |
|  | rs11600716 | G | C | No Data | *MS4A2;LOC105369319* | downstream | chr11 | 59924421 | 6 | 1.57 |
|  | rs920573 | G | A | No Data | *MS4A2;LOC105369319* | downstream | chr11 | 59924958 | No Data | 1.14 |
|  | rs17529983 | G | A | No Data | *LOC105369319* | nc transcript variant | chr11 | 59927038 | 5 | 1.16 |
|  | rs11605427 | G | C | No Data | *LOC105369319* | intron variant,  nc transcript variant | chr11 | 59928671 | 3a | 1.18 |
|  | rs7926954 | G | A | No Data | *LOC105369319* | upstream variant 2KB | chr11 | 59932578 | No Data | 1.28 |
|  | rs56189574 | A | G | No Data | *LOC105369319* | upstream variant 2KB | chr11 | 59933491 | 4 | 1.88 |
|  | rs7933202 | A | C | No Data | *LOC105369319;*  *MS4A6A* | intergenic | chr11 | 59936925 | 2b | 62.21 |
|  | **rs610932** | T | G | ? | *MS4A6A* | downstream variant500B,  utr variant 3 prime | chr11 | 59939306 | No Data | 1.08 |
|  | rs7935829 | A | G | No Data | *MS4A6A* | intron variant | chr11 | 59942814 | 6 | 1.65 |
|  | rs72918674 | C | T | No Data | *MS4A6A* | intron variant,  utr variant 3 prime | chr11 | 59944579 | No Data | 1.07 |
|  | rs7946992 | T | C | No Data | *MS4A6A* | intron variant,  utr variant 3 prime | chr11 | 59944809 | No Data | 1.44 |
|  | rs12453 | T | C | No Data | *MS4A6A* | synonymous codon | chr11 | 59945744 | 5 | 0.97 |
|  | **rs583791** | C | T | No Data | *MS4A6A* | intron variant | chr11 | 59947251 | No Data | 3.29 |
|  | rs17602572 | C | G | No Data | *MS4A6A* | intron variant | chr11 | 59948373 | 1f | 1.66 |
|  | rs2081545 | C | A | No Data | *MS4A6A;MS4A4E* | intergenic | chr11 | 59958379 | No Data | 1.26 |
|  | rs1834550 | T | C | No Data | *MS4A6A;MS4A4E* | intergenic | chr11 | 59959842 | No Data | 1.25 |
|  | rs10897011 | G | A | No Data | *MS4A6A;MS4A4E* | intergenic | chr11 | 59961426 | 4 | 105.58 |
|  | rs11230180 | G | T | No Data | *MS4A6A;MS4A4E* | intergenic | chr11 | 59961485 | 1f | 25.4 |
|  | rs7926729 | C | T | No Data | *MS4A6A;MS4A4E* | intergenic | chr11 | 59961785 | 4 | 33.09 |
|  | rs7926344 | G | A | No Data | *MS4A6A;MS4A4E* | intergenic | chr11 | 59962165 | 3a | 9.7 |
|  | rs7926354 | G | A | No Data | *MS4A6A;MS4A4E* | intergenic | chr11 | 59962188 | 4 | 8.04 |
|  | rs7116190 | G | A | No Data | *MS4A6A;MS4A4E* | intergenic | chr11 | 59964991 | 6 | 1.4 |
|  | rs11230183 | C | T | No Data | *MS4A6A;MS4A4E* | intergenic | chr11 | 59965724 | 6 | 0.93 |
|  | rs11230184 | G | A | No Data | *MS4A6A;MS4A4E* | intergenic | chr11 | 59965788 | No Data | 1.08 |
|  | rs2123314 | T | C | No Data | *MS4A6A;MS4A4E* | intergenic | chr11 | 59966294 | 1f | 0.88 |
|  | rs7933805 | A | G | No Data | *MS4A6A;MS4A4E* | intergenic | chr11 | 59967525 | 5 | 0.86 |
|  | **rs1582763** | G | A | G | *MS4A4E ;MS4A4A* | intergenic | chr11 | 60021947 | 1f | 3.44 |
|  | **rs1562990** | C | A | ? | *MS4A4E ;MS4A4A* | intergenic | chr11 | 60023086 | 6 | 0.97 |
|  | **rs4938933** | C | T | ? | *MS4A4E ;MS4A4A* | intergenic | chr11 | 60034428 | No Data | 1.56 |
| **11q14** | **rs1385600** | A | G | No Data | *GAB2* | nc transcript variant,  synonymous codon | chr11 | 77936165 | 4 | 1.57 |
|  | **rs4945261** | G | A | No Data | *GAB2* | intron variant | chr11 | 77990259 | 5 | 2.58 |
|  | **rs2373115** | C | A | G | *GAB2* | intron variant | chr11 | 78091149 | No Data | 5.43 |
|  | **rs12798898** | A | G | No Data | *CCDC83;PICALM* | downstream | chr11 | 85659579 | 5 | 24.49 |
|  | **rs17817600** | A | G | ? | *PICALM* | intron variant | chr11 | 85677470 | 5 | 1.77 |
|  | **rs10501602** | A | G | No Data | *PICALM* | intron variant | chr11 | 85681388 | 6 | 1.55 |
|  | **rs17817648** | T | C | No Data | *PICALM* | intron variant | chr11 | 85685284 | 4 | 3.12 |
|  | **rs536841** | C | T | ? | *PICALM;FNTAP1* | upstream | chr11 | 85787823 | 5 | 1.26 |
|  | **rs541458** | C | T | No Data | *PICALM;FNTAP1* | upstream | chr11 | 85788350 | 5 | 1.88 |
|  | **rs561655** | G | A | ? | *PICALM;FNTAP1* | upstream | chr11 | 85800278 | 5 | 3.36 |
|  | **rs543293** | A | G | No Data | *PICALM;FNTAP1* | upstream | chr11 | 85820076 | 4 | 1.68 |
|  | **rs471470** | C | A | A | *PICALM;FNTAP1* | upstream | chr11 | 85831540 | 6 | 1.5 |
|  | **rs10792832** | A | G | G | *PICALM;FNTAP1* | upstream | chr11 | 85867874 | 3a | 6.26 |
|  | **rs3851179** | T | C | ? | *PICALM;FNTAP1* | upstream | chr11 | 85868639 | 6 | 2.93 |
| **11q22** | rs526215 | C | T | No Data | *WTAPP1* | intron variant | chr11 | 102678437 | No Data | 2.89 |
|  | rs1144396 | C | A | No Data | *WTAPP1* | intron variant | chr11 | 102679051 | 4 | 83.59 |
|  | rs502174 | A | C | No Data | *WTAPP1* | intron variant | chr11 | 102682502 | 5 | 1.36 |
|  | rs2097248 | C | T | No Data | *WTAPP1* | intron variant | chr11 | 102690500 | 4 | 2.91 |
|  | rs2408525 | T | G | No Data | *WTAPP1* | intron variant | chr11 | 102690601 | No Data | 1.53 |
|  | rs756544 | G | C | No Data | *WTAPP1* | intron variant | chr11 | 102691161 | No Data | 1.35 |
|  | rs12804136 | G | A | No Data | *WTAPP1* | intron variant | chr11 | 102691389 | 6 | 5.02 |
|  | rs11225434 | T | C | No Data | *WTAPP1* | intron variant | chr11 | 102691481 | 5 | 1.24 |
|  | rs7117013 | A | G | No Data | *WTAPP1* | intron variant | chr11 | 102692223 | 6 | 1.39 |
|  | rs10895364 | C | T | No Data | *WTAPP1* | intron variant | chr11 | 102692862 | No Data | 2.46 |
|  | rs12801529 | T | A | No Data | *WTAPP1* | intron variant | chr11 | 102693609 | No Data | 1.92 |
|  | rs10895365 | C | T | No Data | *WTAPP1* | intron variant | chr11 | 102694192 | 6 | 4.56 |
|  | rs10895366 | G | A | No Data | *WTAPP1* | intron variant | chr11 | 102697088 | 5 | 1.55 |
|  | rs7126392 | G | A | No Data | *WTAPP1* | intron variant | chr11 | 102697120 | 5 | 3.16 |
|  | rs12792871 | C | G | No Data | *WTAPP1* | intron variant | chr11 | 102698335 | No Data | 1.15 |
|  | rs7926920 | G | A | No Data | *WTAPP1* | intron variant | chr11 | 102698723 | 6 | 1.24 |
|  | rs7946057 | A | T | No Data | *WTAPP1* | intron variant | chr11 | 102699459 | 6 | 1.54 |
|  | rs1010698 | G | T | No Data | *WTAPP1* | intron variant | chr11 | 102701020 | 5 | 3.31 |
|  | rs2155013 | T | C | No Data | *WTAPP1* | intron variant | chr11 | 102701857 | 6 | 2.75 |
|  | rs4754884 | G | A | No Data | *WTAPP1* | intron variant | chr11 | 102702262 | 4 | 95.1 |
|  | rs635746 | A | G | No Data | *WTAPP1* | nc transcript | chr11 | 102703627 | No Data | 1.72 |
|  | rs633962 | C | T | No Data | *WTAPP1* | intron variant | chr11 | 102704070 | No Data | 1.57 |
|  | rs666734 | G | C | No Data | *WTAPP1* | intron variant | chr11 | 102704179 | No Data | 1.77 |
|  | rs595840 | A | G | No Data | *WTAPP1* | intron variant | chr11 | 102704896 | 6 | 1.51 |
|  | rs639752 | C | A | No Data | *MMP3;WTAPP1* | intron variant,  nc transcript variant | chr11 | 102707338 | 4 | 18.24 |
|  | rs575027 | A | G | No Data | *MMP3;WTAPP1* | downstream variant 500B,  intron variant | chr11 | 102707913 | No Data | 1.79 |
|  | rs520540 | A | G | No Data | *MMP3* | synonymous codon | chr11 | 102709424 | No Data | 1.67 |
|  | rs591058 | T | C | No Data | *MMP3* | intron variant | chr11 | 102711337 | 6 | 1.6 |
|  | rs602128 | A | G | No Data | *MMP3* | synonymous codon | chr11 | 102713464 | No Data | 1.18 |
|  | rs679620 | T | C | No Data | *MMP3* | missense | chr11 | 102713619 | No Data | 1.42 |
|  | rs678815 | G | C | No Data | *MMP3* | intron variant | chr11 | 102713776 | 5 | 1.18 |
|  | rs617819 | C | G | No Data | *MMP3* | upstream variant 2KB | chr11 | 102714715 | 6 | 3.98 |
|  | rs632478 | T | G | No Data | *MMP3* | upstream variant 2KB | chr11 | 102715680 | No Data | 1.18 |
|  | rs645419 | A | G | No Data | *MMP3* | upstream variant 2KB | chr11 | 102716320 | No Data | 1.31 |
|  | **rs573521** | A | G | A | *MMP12;MMP3* | intergenic | chr11 | 102716979 | 4 | 2.11 |
| **11q23.2-q24.2** | **rs11218343** | T | C | T | *SORL1* | intron variant,  upstream variant 2KB | chr11 | 121435586 | 5 | 2.52 |
| **12q14** | rs1026025 | A | G | No Data | *WIF1* | intron variant | chr12 | 65449070 | 5 | 2.54 |
|  | rs1446528 | A | G | No Data | *WIF1* | intron variant | chr12 | 65463646 | 6 | 1.08 |
|  | rs2034262 | T | C | No Data | *WIF1* | intron variant | chr12 | 65465613 | 6 | 1.97 |
|  | rs969028 | G | C | No Data | *WIF1* | intron variant | chr12 | 65467576 | No Data | 1.66 |
|  | rs61924763 | C | G | No Data | *WIF1* | intron variant | chr12 | 65478509 | 3a | 4.22 |
|  | rs1446530 | C | A | No Data | *WIF1* | intron variant | chr12 | 65479503 | 5 | 2.68 |
|  | rs1979034 | G | A | No Data | *WIF1* | intron variant | chr12 | 65480037 | 5 | 2.73 |
|  | rs1979035 | A | G | No Data | *WIF1* | intron variant | chr12 | 65480358 | 5 | 2.33 |
|  | rs11612023 | C | T | No Data | *WIF1* | intron variant | chr12 | 65483850 | 6 | 1.39 |
|  | rs59756710 | A | G | No Data | *WIF1* | intron variant | chr12 | 65487615 | 5 | 3.51 |
|  | rs61924764 | A | G | No Data | *WIF1* | intron variant | chr12 | 65488851 | 6 | 2.19 |
|  | rs61924765 | C | A | No Data | *WIF1* | intron variant | chr12 | 65493281 | 3a | 4.37 |
|  | rs61924766 | G | C | No Data | *WIF1* | intron variant | chr12 | 65496008 | No Data | 1.02 |
|  | rs17765135 | C | G | No Data | *WIF1* | intron variant | chr12 | 65503746 | 6 | 1.25 |
|  | rs4762126 | T | C | No Data | *WIF1* | intron variant | chr12 | 65504923 | 6 | 3.09 |
|  | rs10878230 | T | G | No Data | *WIF1* | intron variant | chr12 | 65508787 | No Data | 1.01 |
|  | rs2173456 | G | A | No Data | *WIF1* | intron variant | chr12 | 65511351 | 6 | 1.02 |
|  | rs7301320 | T | C | No Data | *WIF1* | synonymous codon | chr12 | 65514265 | No Data | 1.17 |
|  | rs10878232 | T | G | No Data | *LEMD3;WIF1* | upstream | chr12 | 65522646 | 6 | 1.2 |
|  | **rs1155722** | T | C | No Data | *LEMD3;WIF1* | upstream | chr12 | 65523156 | 6 | 0.98 |
|  | rs6581611 | T | C | No Data | *LEMD3;WIF1* | upstream | chr12 | 65526125 | No Data | 1.05 |
|  | rs2336435 | A | G | No Data | *LEMD3;WIF1* | upstream | chr12 | 65527886 | 6 | 0.8 |
|  | rs10784439 | C | T | No Data | *LEMD3;WIF1* | upstream | chr12 | 65533855 | No Data | 1.54 |
|  | **rs6581612** | C | A | No Data | *LEMD3;WIF1* | upstream | chr12 | 65534623 | 5 | 0.81 |
|  | **rs10784440** | C | T | No Data | *LEMD3;WIF1* | upstream | chr12 | 65536606 | 6 | 1.33 |
|  | **rs1498792** | T | C | No Data | *LEMD3;WIF1* | upstream | chr12 | 65537669 | 6 | 1.25 |
|  | **rs2047485** | G | A | No Data | *LEMD3;WIF1* | upstream | chr12 | 65538010 | No Data | 1.14 |
|  | **rs1498793** | A | G | No Data | *LEMD3;WIF1* | upstream | chr12 | 65539421 | 6 | 0.84 |
|  | **rs7302095** | C | A | No Data | *LEMD3;WIF1* | upstream | chr12 | 65540793 | 6 | 0.95 |
|  | **rs7307410** | C | G | No Data | *LEMD3;WIF1* | upstream | chr12 | 65542577 | No Data | 0.9 |
|  | **rs7138233** | T | C | No Data | *LEMD3;WIF1* | upstream | chr12 | 65542653 | No Data | 0.9 |
|  | rs1391742 | A | G | No Data | *LEMD3;WIF1* | upstream | chr12 | 65546673 | 5 | 2.15 |
|  | **rs7309669** | G | T | No Data | *LEMD3;WIF1* | upstream | chr12 | 65547820 | No Data | 0.87 |
|  | rs10748017 | C | T | No Data | *LEMD3;WIF1* | upstream | chr12 | 65548535 | 6 | 0.98 |
|  | **rs6581613** | A | G | No Data | *LEMD3;WIF1* | upstream | chr12 | 65549130 | 6 | 0.78 |
|  | rs7137979 | G | C | No Data | *LEMD3;WIF1* | upstream | chr12 | 65551428 | No Data | 1.48 |
|  | **rs7308958** | T | C | No Data | *LEMD3;WIF1* | upstream | chr12 | 65551526 | 5 | 1.01 |
|  | rs17177768 | C | T | No Data | *LEMD3;WIF1* | upstream | chr12 | 65552793 | 5 | 1.94 |
|  | rs7300703 | G | A | No Data | *LEMD3;WIF1* | upstream | chr12 | 65552860 | 5 | 0.84 |
|  | rs6581615 | A | G | No Data | *LEMD3;WIF1* | upstream | chr12 | 65556178 | No Data | 1.22 |
|  | rs11175696 | A | C | No Data | *LEMD3* | intron variant | chr12 | 65638064 | 6 | 5.09 |
|  | **rs17178006** | T | G | No Data | *MSRB3* | intron variant | chr12 | 65718298 | 5 | 4.93 |
|  | rs73123652 | T | C | No Data | *LOC100507065;*  *LOC105369809* | intron variant | chr12 | 65874955 | 6 | 1.39 |
| **12q22** | **rs61144803** | A | G | ? | *CRADD* | intron variant | chr12 | 94235164 | No Data | 1.2 |
| **12q24** | rs7315280 | A | G | No Data | *HRK* | upstream variant 2KB | chr12 | 117320937 | 4 | 64.14 |
|  | rs7137149 | T | C | No Data | *HRK;FBXW8* | upstream | chr12 | 117321661 | No Data | 2.29 |
|  | rs77956314 | T | C | No Data | *HRK;FBXW8* | upstream | chr12 | 117323366 | 3a | 2.77 |
|  | rs11068224 | G | A | No Data | *HRK;FBXW8* | upstream | chr12 | 117324966 | No Data | 0.92 |
|  | rs12311895 | T | C | No Data | *HRK;FBXW8* | upstream | chr12 | 117326926 | 3a | 2.13 |
|  | rs113205216 | C | A | No Data | *HRK;FBXW8* | upstream | chr12 | 117326942 | 3a | 2.25 |
|  | rs113179672 | G | A | No Data | *HRK;FBXW8* | upstream | chr12 | 117327240 | 5 | 2.97 |
|  | **rs7294919** | T | C | No Data | *FBXW8;HRK* | upstream | chr12 | 117327591 | 6 | 1.7 |
|  | rs7305705 | G | T | No Data | *HRK;FBXW8* | upstream | chr12 | 117327650 | 6 | 1.95 |
|  | rs10850728 | T | C | No Data | *HRK;FBXW8* | upstream | chr12 | 117330482 | 4 | 7.64 |
|  | rs11836634 | A | G | No Data | *HRK;FBXW8* | upstream | chr12 | 117330843 | 5 | 1.51 |
|  | rs111865233 | T | C | No Data | *HRK;FBXW8* | upstream | chr12 | 117332155 | 5 | 1.22 |
|  | **rs7312900** | A | T | No Data | *FBXW8;HRK* | upstream | chr12 | 117332369 | 5 | 1.25 |
|  | rs12313220 | T | C | No Data | *HRK;FBXW8* | upstream | chr12 | 117332880 | 6 | 1.86 |
|  | rs7309079 | G | C | No Data | *HRK;FBXW8* | upstream | chr12 | 117333977 | 6 | 1.03 |
|  | rs76845667 | C | T | No Data | *HRK;FBXW8* | upstream | chr12 | 117334486 | 4 | 2.49 |
|  | **rs12310299** | C | G | No Data | *HRK;FBXW8* | upstream | chr12 | 117335486 | 5 | 0.95 |
|  | rs7131959 | G | A | No Data | *HRK;FBXW8* | upstream | chr12 | 117335614 | No Data | 1.03 |
|  | rs80001826 | G | C | No Data | *HRK;FBXW8* | upstream | chr12 | 117335651 | 6 | 0.98 |
|  | **rs7132339** | G | A | No Data | *FBXW8;HRK* | upstream | chr12 | 117335911 | 1b | 3.01 |
|  | **rs7133290** | C | T | No Data | *FBXW8;HRK* | upstream | chr12 | 117335943 | 1f | 1.41 |
|  | rs113844630 | T | C | No Data | *HRK;FBXW8* | upstream | chr12 | 117337102 | No Data | 1.73 |
|  | rs113315762 | T | C | No Data | *HRK;FBXW8* | upstream | chr12 | 117337236 | No Data | 1.37 |
|  | rs75337356 | T | C | No Data | *HRK;FBXW8* | upstream | chr12 | 117337253 | No Data | 0.97 |
|  | rs12302173 | G | A | No Data | *HRK;FBXW8* | upstream | chr12 | 117338503 | 6 | 2.21 |
|  | rs117425351 | A | G | No Data | *FBXW8* | intron variant | chr12 | 117409751 | No Data | 1.1 |
|  | rs118117321 | C | G | No Data | *FBXW8;LOC100506551* | intron variant,  downstream variant 500B | chr12 | 117414789 | 4 | 1.96 |
|  | **rs1997111** | A | C | No Data | *LOC105370020* | intron variant | chr12 | 119387917 | No Data | 2.04 |
| **13q12** | rs7332961 | C | T | No Data | *LHFP* | intron variant | chr13 | 40114862 | No Data | 1.97 |
|  | rs9532396 | T | C | No Data | *LHFP* | intron variant | chr13 | 40117270 | 6 | 1.84 |
|  | rs9315701 | G | A | No Data | *LHFP* | intron variant | chr13 | 40117991 | 5 | 2.21 |
|  | **rs9315702** | C | A | A | *LHFP* | intron variant | chr13 | 40118067 | 5 | 1.88 |
|  | rs2324337 | A | G | No Data | *LHFP* | intron variant | chr13 | 40118863 | No Data | 1.93 |
|  | rs9548804 | T | G | No Data | *LHFP* | intron variant | chr13 | 40121426 | 6 | 2.99 |
|  | rs9548805 | G | T | No Data | *LHFP* | intron variant | chr13 | 40121660 | 6 | 2.1 |
|  | rs9548809 | T | C | No Data | *LHFP* | intron variant | chr13 | 40123504 | No Data | 2.68 |
|  | rs9548819 | A | T | No Data | *LHFP* | intron variant | chr13 | 40128302 | 6 | 1.94 |
|  | rs9315703 | T | C | No Data | *LHFP* | intron variant | chr13 | 40128844 | 5 | 5.37 |
|  | rs9548820 | G | A | No Data | *LHFP* | intron variant | chr13 | 40129211 | No Data | 1.84 |
|  | rs7982917 | G | A | No Data | *LHFP* | intron variant | chr13 | 40132276 | 6 | 1.88 |
|  | rs9548821 | A | G | No Data | *LHFP* | intron variant | chr13 | 40132990 | 6 | 1.71 |
|  | rs9548822 | T | C | No Data | *LHFP* | intron variant | chr13 | 40135449 | No Data | 2.29 |
|  | rs7981547 | T | C | No Data | *LHFP* | intron variant | chr13 | 40136246 | 5 | 7.87 |
|  | rs35396912 | C | T | No Data | *LHFP* | intron variant | chr13 | 40136851 | 5 | 9.28 |
|  | rs9548824 | C | G | No Data | *LHFP* | intron variant | chr13 | 40137487 | 5 | 21.08 |
|  | rs9548825 | T | G | No Data | *LHFP* | intron variant | chr13 | 40138007 | 6 | 32.06 |
|  | rs9532399 | C | T | No Data | *LHFP* | intron variant | chr13 | 40138670 | No Data | 34.27 |
|  | rs9603564 | A | G | No Data | *LHFP* | intron variant | chr13 | 40140701 | 5 | 20.88 |
|  | rs9315706 | G | C | No Data | *LHFP* | intron variant | chr13 | 40141312 | 3a | 7.33 |
|  | rs9548826 | C | A | No Data | *LHFP* | intron variant | chr13 | 40141791 | 5 | 12.75 |
|  | rs9548827 | G | A | No Data | *LHFP* | intron variant | chr13 | 40141814 | 5 | 12.51 |
|  | rs7322336 | G | A | No Data | *LHFP* | intron variant | chr13 | 40144948 | 6 | 8.47 |
|  | rs9315707 | T | C | No Data | *LHFP* | intron variant | chr13 | 40145283 | No Data | 2.04 |
|  | rs1885759 | C | T | No Data | *LHFP* | intron variant | chr13 | 40145423 | No Data | 1.97 |
|  | rs9548829 | G | A | No Data | *LHFP* | intron variant | chr13 | 40146052 | No Data | 5.63 |
|  | rs9548830 | C | A | No Data | *LHFP* | intron variant | chr13 | 40146468 | 5 | 1.93 |
|  | rs7333056 | A | G | No Data | *LHFP* | intron variant | chr13 | 40148180 | 6 | 2.05 |
| **13q33** | **rs17393344** | G | A | ? | *MYO16* | intron variant | chr13 | 109473945 | 5 | 1.09 |
| **14q22** | rs17125924 | A | G | No Data | *FERMT2;*  *LOC105370500* | intron variant | chr14 | 53391679 | No Data | 10.27 |
|  | **rs17125944** | T | C | C | *FERMT2* | intron variant | chr14 | 53400628 | 5 | 6.29 |
| **14q32** | **rs10498633** | G | T | G | *SLC24A4* | intron variant | chr14 | 92926951 | 5 | 12.29 |
|  | rs12881735 | T | C | No Data | *SCL24A4* | intron variant | chr14 | 92932827 | 6 | 1.19 |
|  | rs36026988 | T | C | No Data | *SCL24A4* | intron variant | chr14 | 92938381 | 4 | 38.89 |
|  | **rs115102486** | A | G | ? | *CLMN* | intron variant | chr14 | 95764563 | 5 | 4.16 |
| **16p12** | **rs8045064** | T | C | ? | *LINC01567* | intron variant | chr16 | 24675588 | No Data | 1.1 |
| **17q21** | **rs2732703** |  |  | T | *ARL17A;ARL17B;*  *LRRC37A2;LRRC37A* | intron variant | chr17 | 44353221 | 5 | 1.4 |
| **17q22** | **rs117964204** | C | T | ? | *CACNA1G* | intron variant | chr17 | 48692081 | 2b | 5.53 |
| **17q23** | **rs72832584** | A | C | ? | *BCAS3* | intron variant | chr17 | 59292435 | 5 | 0.9 |
|  | **rs4968782** | G | A | G | *ACE;CYB561* | upstream | chr17 | 61548475 | No Data | 1.26 |
| **19p13** | **rs3764650** | T | G | ? | *ABCA7* | intron variant,  upstreamvariant2KB | chr19 | 1046519 | 2a | 7.8 |
|  | **rs115550680** | A | G | G | *ABCA7* | intron variant | chr19 | 1050419 | 5 | 3.2 |
|  | **rs4147929** | A | G | A | *ABCA7* | intron variant | chr19 | 1063442 | 4 | 8.41 |
| **19q13** | **rs714948** | C | A | No Data | *PVR* | utr variant 3 prime | chr19 | 45165911 | 5 | 8.49 |
|  | **rs2965109** | C | T | No Data | *LOC105372416* | intron variant | chr19 | 45225344 | 4 | 26.6 |
|  | **rs7254776** | T | C | No Data | *LOC105372416* | intron variant | chr19 | 45227741 | 4 | 49.18 |
|  | **rs1551891** | G | A | No Data | *LOC105372416* | intron variant | chr19 | 45231820 | No Data | 6.7 |
|  | **rs62117161** | A | G | No Data | *LOC105372416* | intron variant | chr19 | 45233384 | No Data | 5.86 |
|  | **rs2965101** | T | C | No Data | *LOC105372416* | intron variant | chr19 | 45237811 | No Data | 5.22 |
|  | **rs62117162** | C | A | No Data | *LOC105372416* | intron variant | chr19 | 45239535 | No Data | 5.34 |
|  | **rs2927438** | A | G | No Data | *LOC105372416* | intron variant | chr19 | 45242106 | 3a | 34.78 |
|  | **rs1531517** | G | A | No Data | *LOC105372416* | intron variant | chr19 | 45242172 | 4 | 36.85 |
|  | **rs62117204** | C | T | No Data | *LOC105372416* | upstream variant 2KB | chr19 | 45242966 | No Data | 16.22 |
|  | **rs8100239** | T | A | No Data | *BCL3* | intron variant,  upstreamvariant2KB | chr19 | 45253103 | 4 | 68.4 |
|  | **rs8103315** | C | A | No Data | *BCL3* | intron variant | chr19 | 45254167 | 2b | 21.7 |
|  | **rs2927477** | T | C | No Data | *BCAM* | intron variant | chr19 | 45313714 | 2b | 28.82 |
|  | **rs10405693** | C | T | No Data | *BCAM;NECTIN2* | intergenic | chr19 | 45326663 | 4 | 20.44 |
|  | **rs4803759** | T | C | No Data | *BCAM;NECTIN2* | intergenic | chr19 | 45327458 | 6 | 8.55 |
|  | **rs10402271** | T | G | No Data | *BCAM;NECTIN2* | intergenic | chr19 | 45329213 | 5 | 8.49 |
|  | **rs4802238** | C | T | No Data | *BCAM;PVRL2* | intergenic | chr19 | 45331724 | No Data | 9.36 |
|  | **rs7359852** | T | C | No Data | *BCAM;NECTIN2* | intergenic | chr19 | 45336034 | 6 | 8.49 |
|  | **rs2927480** | G | C | No Data | *BCAM;NECTIN2* | intergenic | chr19 | 45337384 | No Data | 8.89 |
|  | **rs4605275** | T | C | No Data | *BCAM;NECTIN2* | intergenic | chr19 | 45338492 | 6 | 8.52 |
|  | **rs1871047** | A | G | No Data | *PVRL2* | intron variant | chr19 | 45351745 | 1b | 96.1 |
|  | **rs1871046** | T | C | No Data | *PVRL2* | intron variant | chr19 | 45351936 | 2a | 99.08 |
|  | **rs4803763** | G | C | No Data | *PVRL2* | intron variant | chr19 | 45357290 | 5 | 9.45 |
|  | **rs440277** | G | A | No Data | *PVRL2* | intron variant | chr19 | 45361223 | 1f | 15.52 |
|  | **rs377702** | G | A | No Data | *PVRL2* | intron variant | chr19 | 45362666 | 2b | 12.49 |
|  | **rs12978931** | A | G | No Data | *PVRL2* | intron variant | chr19 | 45363699 | No Data | 8.88 |
|  | **rs519825** | T | C | No Data | *PVRL2* | intron variant | chr19 | 45366778 | 5 | 8.9 |
|  | **rs12610605** | G | A | No Data | *PVRL2* | intron variant | chr19 | 45370837 | 5 | 11.61 |
|  | **rs416041** | G | A | No Data | *PVRL2* | intron variant | chr19 | 45370853 | 5 | 12.32 |
|  | **rs519113** | C | G | G | *PVRL2* | intron variant | chr19 | 45376283 | 1f | 9.67 |
|  | **rs387976** | A | C | No Data | *PVRL2* | intron variant | chr19 | 45379059 | 5 | 21.46 |
|  | **rs11667640** | C | T | No Data | *PVRL2* | intron variant | chr19 | 45379790 | 4 | 23.78 |
|  | **rs6859** | A | G | A | *PVRL2* | intron variant,  utrvariant3prime | chr19 | 45382033 | 4 | 12.26 |
|  | **rs11669338** | T | G | No Data | *PVRL2* | intron variant | chr19 | 45382983 | 4 | 14.62 |
|  | **rs11673139** | A | T | No Data | *PVRL2* | intron variant | chr19 | 45383036 | 4 | 16 |
|  | **rs3852861** | G | T | No Data | *PVRL2* | intron variant | chr19 | 45383060 | 4 | 16.18 |
|  | **rs3745150** | G | C | No Data | *PVRL2* | intron variant | chr19 | 45385758 | 5 | 11.7 |
|  | **rs12972156** | C | G | No Data | *PVRL2* | intron variant | chr19 | 45387458 | 3a | 9.87 |
|  | **rs12972970** | G | A | No Data | *PVRL2* | intron variant | chr19 | 45387595 | No Data | 9.65 |
|  | **rs34342646** | G | A | No Data | *PVRL2* | intron variant | chr19 | 45388129 | No Data | 8.83 |
|  | **rs6857** | C | T | T | *PVRL2* | utr variant 3 prime | chr19 | 45392253 | No Data | 8.87 |
|  | **rs71352238** | T | C | No Data | *TOMM40* | upstreamvariant2KB | chr19 | 45394335 | 4 | 208.63 |
|  | **rs157580** | G | A | ? | *TOMM40* | intron variant | chr19 | 45395265 | 1f | 108.56 |
|  | **rs2075650** | A | G | G | *TOMM40* | intron variant | chr19 | 45395618 | 1f | 62.62 |
|  | **rs34404554** | C | G | No Data | *TOMM40* | intron variant | chr19 | 45395908 | 5 | 45.91 |
|  | **rs11556505** | C | T | No Data | *TOMM40* | synonymous codon | chr19 | 45396143 | 5 | 38.48 |
|  | **rs59007384** | G | T | T | *TOMM40* | intron variant | chr19 | 45396664 | 4 | 13.46 |
|  | **rs8106922** | A | G | No Data | *TOMM40* | intron variant | chr19 | 45401665 | 5 | 3.57 |
|  | **rs1160985** | C | T | No Data | *TOMM40* | intron variant | chr19 | 45403411 | 1f | 3.61 |
|  | **rs760136** | A | G | No Data | *TOMM40* | intron variant | chr19 | 45403857 | 5 | 3.5 |
|  | **rs741780** | T | C | No Data | *TOMM40* | intron variant | chr19 | 45404430 | 5 | 3.51 |
|  | **rs405697** | A | G | No Data | *TOMM40* | intron variant,  synonymous codon | chr19 | 45404690 | 2b | 7.81 |
|  | **rs1038025** | T | C | No Data | *TOMM40* | downstream variant 500B,  intron variant | chr19 | 45404971 | 6 | 3.55 |
|  | **rs1038026** | A | G | No Data | *TOMM40* | downstream variant 500B,  intron variant | chr19 | 45405061 | No Data | 3.4 |
|  | **rs10119** | G | A | No Data | *TOMM40* | utr variant 3 prime | chr19 | 45406672 | 3a | 11.41 |
|  | **rs7259620** | G | A | No Data | *APOE* | upstream variant 2KB | chr19 | 45407787 | 4 | 27.1 |
|  | **rs405509** | T | G | No Data | *APOE* | upstreamvariant2KB | chr19 | 45408835 | 1f | 49.34 |
|  | **rs769449** | G | A | A | *APOE* | intron variant | chr19 | 45410001 | 4 | 28.94 |
|  | **rs429358** | T | C | ? | *APOE* | missense | chr19 | 45411940 | 5 | 4.61 |
|  | **rs439401** | T | C | ? | *APOE;APOC1* | non coding transcript  exon_variant | chr19 | 45414450 | 1b | 152.3 |
|  | **rs56131196** | G | A | A | *APOC1* | downstream variant 500B | chr19 | 45422845 | No Data | 3.9 |
|  | **rs4420638** | A | G | ? | *APOC1* | downstream variant 500B | chr19 | 45422945 | 5 | 3.77 |
|  | **rs4803770** | C | G | No Data | *APOC1;APOC1P1* | intergenic | chr19 | 45427352 | 4 | 4.33 |
|  | **rs17643262** | G | A | No Data | *PPP1R37* | intron variant | chr19 | 45631815 | 4 | 4.11 |
|  | **rs1114832** | C | T | No Data | *PPP1R37* | intron variant | chr19 | 45636200 | 6 | 3.37 |
|  | **rs1114831** | C | A | No Data | *PPP1R37* | intron variant | chr19 | 45636318 | No Data | 3.57 |
|  | **rs1048699** | C | T | No Data | *PPP1R37* | utr variant 3 prime | chr19 | 45650385 | 5 | 5.12 |
|  | **rs10416371** | A | C | No Data | *LOC105372420;NKPD1* | intron variant | chr19 | 45660135 | 4 | 7.43 |
|  | rs61079153 | T | G | No Data | *EXOC3L2* | intron variant | chr19 | 45724960 | No Data | 2.27 |
|  | rs60269219 | C | T | No Data | *EXOC3L2* | intron variant | chr19 | 45724962 | No Data | 2.33 |
|  | rs73568222 | A | G | No Data | *EXOC3L2* | intron variant | chr19 | 45725974 | 5 | 2.31 |
|  | **rs10422797** | T | C | No Data | *EXOC3L2* | intron variant | chr19 | 45726105 | 5 | 2.41 |
|  | rs10423031 | T | G | No Data | *EXOC3L2* | intron variant | chr19 | 45726223 | 5 | 3.34 |
|  | rs114034486 | A | G | No Data | *EXOC3L2* | intron variant | chr19 | 45727275 | 5 | 2.38 |
|  | rs10403626 | C | T | No Data | *EXOC3L2* | intron variant | chr19 | 45727670 | 5 | 4.06 |
|  | rs35087817 | C | T | No Data | *EXOC3L2* | intron variant | chr19 | 45729199 | 6 | 2.29 |
|  | rs346763 | G | A | No Data | *EXOC3L2* | intron variant | chr19 | 45729274 | No Data | 3.48 |
|  | rs8109063 | A | C | No Data | *EXOC3L2* | intron variant | chr19 | 45729586 | 5 | 3.05 |
|  | rs56187099 | G | A | No Data | *EXOC3L2* | intron variant | chr19 | 45731563 | 5 | 4.02 |
|  | **rs3865444** | C | A | ? | *CD33* | nctranscriptvariant,upstreamvariant2KB,utrvariant5prime | chr19 | 51727961 | 5 | 2.87 |
| **20p13** | **rs34972666** | A | G | ? | *TGM6* | intron variant | chr20 | 2384971 | 6 | 1.66 |
| **20q11** | **rs6087664** | C | G | No Data | *TRPC4AP* | intron variant | chr20 | 33626215 | 6 | 5.25 |
|  | **rs6088692** | A | G | No Data | *TRPC4AP* | intron variant | chr20 | 33638587 | 6 | 7.12 |
|  | **rs6120816** | G | C | No Data | *TRPC4AP* | intron variant,  upstream variant 2KB | chr20 | 33644357 | 6 | 8.62 |
|  | **rs1885119** | T | C | No Data | *TRPC4AP* | upstream variant 3KB | chr20 | 33645648 | 6 | 5.11 |
|  | **rs2065108** | C | T | No Data | *EDEM2* | intron variant | chr20 | 33706821 | 1f | 3.62 |
|  | **rs6088727** | G | A | No Data | *EDEM2* | intron variant | chr20 | 33713638 | 1f | 1.87 |
| **20q13** | rs73156368 | C | T | No Data | *CASS4* | intron variant | chr20 | 54988071 | 5 | 32.25 |
|  | rs6069737 | C | T | No Data | *CASS4* | intron variant | chr20 | 54995698 | No Data | 13.93 |
|  | rs6024870 | G | A | No Data | *CASS4* | intron variant | chr20 | 54997567 | 2b | 79.72 |
|  | rs6014724 | A | G | No Data | *CASS4* | intron variant | chr20 | 54998543 | 2b | 12.12 |
|  | rs718022 | G | A | No Data | *CASS4* | intron variant | chr20 | 55003464 | 5 | 23.45 |
|  | rs56012565 | G | A | No Data | *CASS4* | intron variant | chr20 | 55005223 | 5 | 24.02 |
|  | rs79181856 | G | C | No Data | *CASS4* | intron variant | chr20 | 55007953 | No Data | 4.73 |
|  | rs76842328 | T | C | No Data | *CASS4* | intron variant | chr20 | 55012991 | 5 | 5.6 |
|  | rs927174 | A | C | No Data | *CASS4* | intron variant | chr20 | 55015165 | 4 | 49.54 |
|  | **rs7274581** | T | C | T | *CASS4* | intron variant | chr20 | 55018259 | 5 | 4.91 |
|  | rs113902203 | A | G | No Data | *CASS4* | intron variant | chr20 | 55018468 | No Data | 4.34 |
| **Xq21** | **rs5984894** | A | G | No Data | *PCDH11X* | intron variant | chrX | 91393736 | No Data | 2.13 |

Bolded SNP**s** are GWAS lead SNPs
